# Main and epistatic loci studies in soybean for *Sclerotinia sclerotiorum* resistance reveal multiple modes of resistance in multi-environments

**DOI:** 10.1038/s41598-017-03695-9

**Published:** 2017-06-15

**Authors:** Tara C. Moellers, Arti Singh, Jiaoping Zhang, Jae Brungardt, Mehdi Kabbage, Daren S. Mueller, Craig R. Grau, Ashish Ranjan, Damon L. Smith, R. V. Chowda-Reddy, Asheesh K. Singh

**Affiliations:** 1 Department of Agronomy, Iowa State University, Ames, Iowa 50011 United States of America; 2 Department of Plant Pathology, University of Wisconsin-Madison, Madison, Wisconsin 53706 United States of America; 3 Department of Plant Pathology, Iowa State University, Ames, Iowa 50011 United States of America

## Abstract

Genome-wide association (GWAS) and epistatic (GWES) studies along with expression studies in soybean [*Glycine max* (L.) Merr.] were leveraged to dissect the genetics of Sclerotinia stem rot (SSR) [caused by *Sclerotinia sclerotiorum* (Lib.) de Bary], a significant fungal disease causing yield and quality losses. A large association panel of 466 diverse plant introduction accessions were phenotyped in multiple field and controlled environments to: (1) discover sources of resistance, (2) identify SNPs associated with resistance, and (3) determine putative candidate genes to elucidate the mode of resistance. We report 58 significant main effect loci and 24 significant epistatic interactions associated with SSR resistance, with candidate genes involved in a wide range of processes including cell wall structure, hormone signaling, and sugar allocation related to plant immunity, revealing the complex nature of SSR resistance. Putative candidate genes [for example, *PHYTOALEXIN DEFFICIENT 4* (*PAD4*), *ETHYLENE-INSENSITIVE 3-LIKE 1* (*EIL3*), and *ETHYLENE RESPONSE FACTOR 1* (*ERF1*)] clustered into salicylic acid (SA), jasmonic acid (JA), and ethylene (ET) pathways suggest the involvement of a complex hormonal network typically activated by both necrotrophic (ET/JA) and biotrophic (SA) pathogens supporting that *S*. *sclerotiorum* is a hemibiotrophic plant pathogen.

## Introduction

Sclerotinia stem rot (SSR) [caused by *Sclerotinia sclerotiorum* (Lib.) de Bary] is a significant disease affecting soybean [*Glycine max* (L.) Merr.]. In addition to soybean, *Sclerotinia sclerotiorum* is reported to infect up to 408 different species^1, 2^. The majority of these species are dicotyledons, although infection on monocotyledon species has been reported^1^. *S*. *sclerotiorum* is predominately a necrotrophic fungus that spreads throughout the main stem causing bleaching and shredding of tissue and severe wilting^3^. SSR is particularly prevalent in cool (<25 °C) moist environments. Since its first report in the United States in 1924, SSR has since spread across the soybean growing regions of Northern United States and Canada^4^. In 2009, SSR was ranked the second most damaging soybean disease in the United States^5^. After high temperatures and drought conditions in the Northern United States in 2012, estimated soybean yield suppression by SSR has gradually increased from 99.1 million kg to 947.4 million kg in 2014 when it was ranked the fourth most damaging disease^6^. In addition to yield losses, SSR may impact seed quality through oil content and reduced germination^7, 8^.

Severity and incidence of SSR is highly variable from year to year due to its sensitivity to weather conditions and the aggregated nature of *S*. *sclerotiorum* in soybean fields, making it difficult to effectively use in-season control measures such as fungicide applications^9^. This makes the development of genetic controls desirable, and great efforts have been made to identify sources of resistance. Since sources of complete resistance have not been identified, sources with partial resistance are used for cultivar development in soybean breeding programs. Identified sources include advanced cultivars, such as NK S19-90, and plant introductions^10, 11^.

Bi-parental linkage mapping has led to the discovery of many quantitative trait loci (QTL) for partial resistance. A total of 103 QTL on 18 of the 20 soybean chromosomes have been recorded on SoyBase^12^ with minimal overlap between QTL reported by different studies. Therefore, they are difficult to utilize in marker assisted breeding.

Genome-wide association (GWAS) approaches overcome the limitations associated with linkage mapping, such as limited allelic segregations between parents and lack of recombination due to population creation, while exploiting historical recombination through high-resolution mapping. GWAS is broadly utilized in soybean in order to dissect the genetic architecture of complex traits^13–16^. GWAS studies have been performed for several biotic stresses including *S*. *sclerotiorum* ^17–19^. However, these studies were done in controlled environments which has been shown to result in poor or inconsistent correlations with field evaluations^11, 20, 21^. Therefore, there is a need for GWAS developed in field and greenhouse environments that complement each other by considering natural host responses and disease conditions. This will enhance the understanding of resistance mechanisms and advance breeding efforts to better accommodate farmer needs.

GWAS has been used to identify many genetic variants associated with diseases; however, these only explain a portion of the heritability of complex traits^22^. A recent study in soybean explored both the additive and epistatic effects associated with sudden death syndrome (SDS) resistance^23^. This was done through both GWAS and genome-wide epistasis studies (GWES), and interactions identified were able to explain additional phenotypic variation. Although GWES has been used in human disease research, it has not been widely used in plants. Since previous studies focused on the additive effects that result from GWAS, information on epistatic interactions could increase the understanding behind the complex genetic architecture of quantitative traits, including the mode of resistance, and better assist breeders in identifying favorable allelic combinations.

The main objectives of this study were to discover new sources of SSR resistance in soybean, identify markers associated with SSR resistance for both additive and epistatic effects, and determine putative candidate genes that control SSR resistance to elucidate the main mode of resistance. In order to achieve this, soybean accessions were evaluated for SSR resistance in various environments. Four hundred sixty-six accessions with available high-density SNP information were used to perform both GWAS and GWES. Disease severity (DS), lesion length (LL), and wilt score (WS) were observed depending on the environment. Putative candidate genes underlying associated loci were used to understand the different mechanisms that underpin SSR resistance in soybean. For further validation, transcript levels of several putative candidate genes were compared in resistant and susceptible breeding lines using RNAseq, and approximately half of the putative candidate genes were differentially expressed.

## Results and Discussion

### Phenotypic variation

Previous efforts to dissect the genetic architecture of SSR resistance in soybean included three GWAS^17–19^. These studies were done in controlled environments for phenotypic expression, but differed in the genetic background (101–330 lines ranging from elite cultivars to landraces) and number of markers (7,864–25,179) used. The present study is the first to phenotype several hundred soybean accessions’ reactions to SSR in multiple greenhouse and field experiments. Experiments were analyzed separately to capture expression of resistant loci in different environments and to develop a more comprehensive understanding of this complex trait.

Under 2014 field conditions (14FLD), the maximum emergence rate was 88.9%, and SSR symptoms were not observed in 61 accessions of the association panel evaluated. Within these 61 accessions, plant emergence numbers varied from 1 to 24 plants (3.7–66.7% emergence). In subsequent experiments, SSR symptoms were observed in all accessions. Disease developed rapidly in young plants inoculated in 2014 greenhouse environments (14GHSE) compared to older plants inoculated in 2015 greenhouse environments (15GHSE). In 2015 field environments (15FLD), methods used to maintain disease promoting conditions included increased canopy density and lengthened irrigation schedule to confer an elongated period of cool, damp conditions. Natural wet weather (Supplementary Fig. S1) favored development of SSR, which increased the frequency of higher severity ratings (Supplementary Fig. S2). These methods included an increase in seeding rate and decreasing the length alleys between plots to develop a denser canopy and inoculating plots in the evening in order to capture an elongated period of cool and damp conditions, aiding in the initial infection of SSR in soybean.

Spearman’s rank correlation coefficients between accessions’ disease responses varied between and among experiments (Supplementary Table S1). Plant DS was evaluated in all experiments at R5, beginning seed growth stage, in field environments, and at three and 14 days after inoculation (DAI) in greenhouse environments. Within an individual greenhouse experiment, correlation coefficients between traits varied from 0.46 to 0.90. In general, field experiments had low correlation coefficients when compared to greenhouse experiments, ranging from 0.12–0.17, except for 14GHSE-DAI14, which were below 0.05.

The low correlations observed in this study suggest that greenhouse experiments, although informative, do not necessarily (or always) correlate with field responses. This observation is supported by previous reports^11, 20, 21, 24–26^. Poor correlations to 14GHSE-DAI14 are most likely due to the early growth stage (V3, third trifoliate), at which inoculation was performed. The rapid spread of disease in this experiment left the bulk of accessions severely wilted or dead by the time of rating. When plants were inoculated at the V5 growth stage (fifth trifoliate) in 15GHSE, the DAI14 values were more informative with higher correlation coefficients between both years of field experiments. Therefore, when using greenhouse screenings, the growth stage at inoculation and response variables measured are vital. With a correlation coefficient of 0.32 (Supplementary Table S1), 14FLD and 15FLD had the highest correlation coefficient between experiments.

Several approaches were used to determine the most resistant accessions for application in genetic enhancement programs: (1) accessions performing equally or better than the resistant check cultivar 93M11 in all environments, (2) performance of the most resistant 10% in field environments, and (3) performance of the most resistant 10% in all environments for all traits. Eleven and 14 soybean accessions were identified from the first two methods, respectively, and PI567264A and PI507491 met the criteria for both (1) and (2) (Supplementary Table S2). Accession PI567264A was the only genotype that met (3). For field resistance, PI227212 is a key accession for field resistance as it ranked in the lowest 1% of both field trials.

### SNP genotyping and genome analysis

A total of 35,683 SNPs distributed over the entire genome were used for association mapping. SNP information was previously prepared using the Illumina Infinium SoySNP50K BeadChip^27^ available through SoyBase^12^. The genome-wide inter-marker distance was 26.6 kb. Chromosome-wide densities varied from 39.2 kb on Chromosome 1 (Gm01) to only 19.9 kb on Gm13. SNPs were also unevenly distributed within chromosomes. The number of SNPs within the euchromatic region (80.2%) is much higher than that found in the heterochromatic region. Since only around 22% of genes are found in the heterochromatic region^28^, the percentage of SNPs within this region is acceptable.

The resolution of association mapping depends on the amount of recombination available, which is measured by linkage disequilibrium (LD) decay rates^29, 30^. LD decay rates were measured separately for euchromatic and heterochromatic regions since about 93% of recombination occurs in euchromatic region in soybean, even though it only accounts for 43% of the genome^28^. When *r* ^2^ reached 0.23, half its maximum value, LD decay rate was estimated at 241 kb and 5061 kb in euchromatic and heterochromatic regions, respectively. This LD decay rate in euchromatic regions was slightly less than previous reports of around 350 kb using similar panels^13, 16^. The average inter-marker distance (26.6 kb) of the SNPs was sufficient to capture the variation within the diverse soybean association panel used in this experiment.

The PI accessions in the association panel had a low heterozygosity rate of 0.6% when 35,683 SNPs with minor allele frequency (MAF) ≥0.05 and missing rate ≤10% were used and reflects the inbreeding nature of soybean. These SNPs had an average nucleotide diversity (polymorphism information content, PIC) of 0.30. This value is between previously reported nucleotide diversity of 0.28 in elite cultivars^31^ and 0.35 in a broad panel of cultivated soybean^32^.

### GWAS analyses

**Figure 1:**
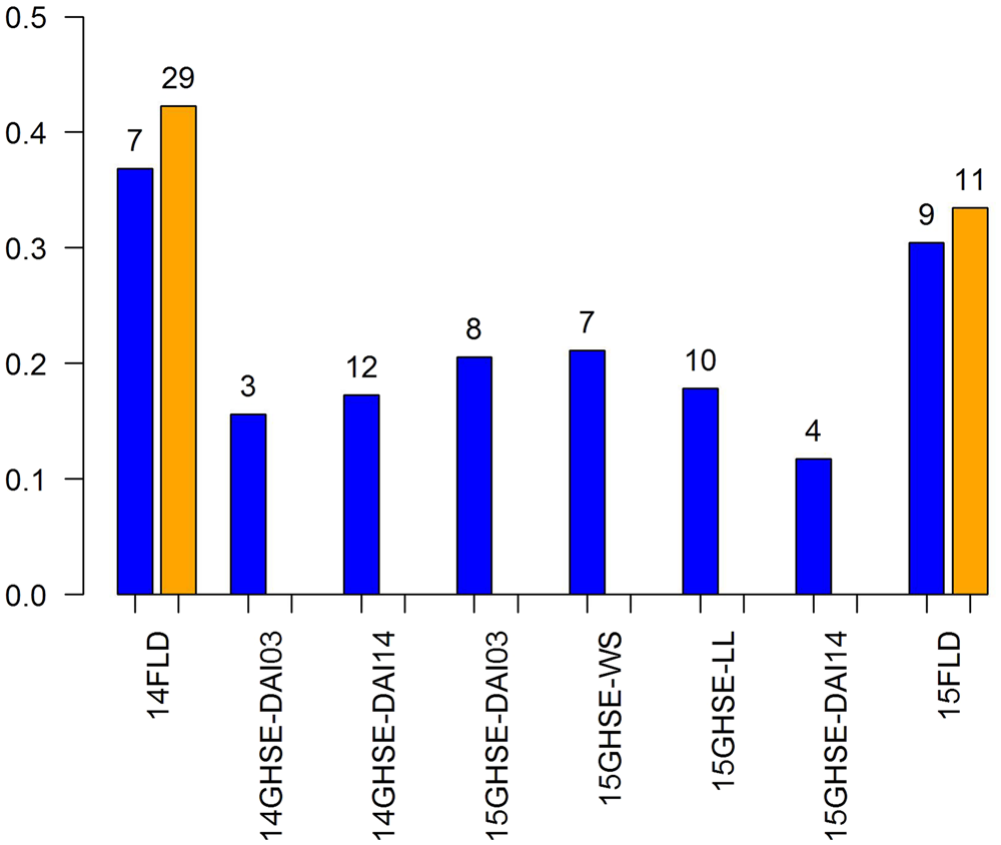
Contributions to the phenotypic variance of identified Sclerotinia stem rot loci via genome-wide association and epistatic analyses for each environment and trait. The number of loci and/or pair of loci used to estimate contributions are indicated above each bar. Blue bars represent contributions due to loci identified using GWAS and orange bars represent contributions of both loci identified using GWAS and loci interactions identified using GWES.

A total of 15, 10, 15, 11, 9, 19, 4, and 15 significant SNPs were associated with 14FLD, 14GHSE-DAI03, 14GHSE-DAI14, 15GHSE-DAI03, 15GHSE-WS, 15GHSE-LL, 15GHSE-DAI14, and 15FLD, respectively. Manhattan plots and QQ plots are presented in Supplementary Figs S3–S6. Significant SNPs found within 50 kb of each side of the strongest trait-associated SNP (peak SNP) were clumped together to form a QTL if contained in an LD block with *r* ^2^ > 0.7 with respect to the peak SNP. The peak SNP was kept to represent the QTL. This condition left a total of 7, 3, 12, 8, 7, 10, 4, and 9 significant loci associated with SSR resistance for 14FLD, 14GHSE-DAI03, 14GHSE-DAI14, 15GHSE-DAI03, 15GHSE-WS, 15GHSE-LL, 15GHSE-DAI14, and 15FLD, respectively (Supplementary Table S3). Due to overlap between traits, 58 significant SNPs were associated with SSR resistance in all experiments. For individual traits, these loci explained 12–37% of the phenotypic variation (Fig. 1). The strongest associated SNP, *ss715607699* was reported for 15GHSE-LL (p-value = 1.69E-06) and 15GHSE-WS (p-value = 8.73E-05). This locus is located within a previously reported QTL^33^.

Overlap between resistant loci were found in 15GHSE. Specifically, two loci were detected for multiple traits whereas both involved WS. Notably, there was no overlap in loci detected between field environments. The lack of overlapping SNPs have been previously reported and discussed^18^. Since our study used the same genotype panel and markers for each experiment, the lack of overlapping SNPs was likely due to a QTL × environment interaction. The lack of correlation between environments can also arise from different underlying mechanisms controlling resistance in different environments. This agrees with quantitatively inherited traits previously reported to display significant environmental interactions^34–36^.

Comparisons for previously reported QTL were done by projecting genetic map locations onto the Glyma. Wm. 82. a2 reference genome physical map^12^. The significant main effect loci from GWAS are presented in Supplementary Table S3. Significant SNPs for SSR resistance were identified on Gm04 and Gm12, where no SSR resistance loci have been reported. The genetic background used in previous studies could have inhibited the detection of loci on these chromosomes in several ways: lack of segregation in bi-parental populations, low allele frequency in the panels chosen for GWAS, or marker density resulting in lack of LD with the causal loci. This study used 466 diverse accessions to increase historic recombination and decrease LD, with over 35 K markers used to capture the genetic variation and increase the genetic resolution. Twenty-one of the 58 identified main effect loci co-localized with previously reported QTL for SSR resistance. Of these, three loci (*ss715599948*, *ss715603406*, and *ss715603408*) located on Gm08 and Gm09 overlapped with multiple QTL previously reported for SSR resistance.

Candidate genes were identified for future functional validation and application. These genes serve as a starting point to better understand the underlying mechanisms of SSR resistance in soybean, but should not be misidentified as causal genes until proper validation. Genes annotated in SoyBase^12^ surrounding the peak SNP served as the source of potential candidate genes. A 50 kB region on either side of the peak SNPs were investigated unless the significant SNPs were involved in a linkage block (*r* ^*2*^ > 0.7 in respect to peak SNP) that resulted in a smaller region to investigate. A total of 57 candidate genes were predicted for main effect loci (Supplementary Table S3). Each candidate gene was assigned a functional category based on its annotations. These functional categories give an idea of the different modes of resistance in response to SSR infection. Approximately, one quarter of the genes were involved in signaling, one quarter in defense, and less than 10% in broader categories such as DNA/RNA (processing, regulation, or organization), protein (synthesis, degradation, modification, or localization), cell wall (synthesis or degradation of cell wall structural proteins), and membrane (structure, transport, or vesicle mediated transport). Some of these functional categories were associated with all experiments, whereas others were limited to a certain experiment. For example, protein candidate genes were only associated with traits measured in 15GHSE, while membrane candidate genes were only specific to 14GHSE-DAI14.

#### Signaling

Putative candidate genes with a signaling functional category included those involved with calcium signaling, G proteins, hormone, and inositol signaling pathway, and general kinases and phosphatases. Signaling genes such as these were differentially expressed between resistant and susceptible soybean genotypes infected with *S*. *sclerotiorum* in a recent expression analysis^37^. The majority of the putative candidate genes identified in the present study were annotated for leucine-rich repeat receptor-like protein kinases (LRR-RLK), one of the major families of plant transmembrane, receptor-like kinases that regulate a wide range of processes, including defense responses^38^.

Putative candidate gene *Glyma*.*06g314000*, associated with 15GHSE-WS, is homologous to *Arabidopsis ETHYLENE-INSENSITIVE 3-LIKE 1* (*EIL1*). *EIL1* is a transcription factor that has been shown to play a key role in the integration of jasmonic acid (JA) and ethylene (ET) signaling, as well as a role in necrotrophic pathogen defense^39^. *Glyma*.*19g248900*, associated with 14FLD, is homologous to *Arabidopsis ETHYLENE RESPONSE FACTOR 1* (*ERF1*). *ERF1* is a transcription factor found downstream of *EIL1* that is induced by ET and/or JA and has an important role in the regulation of defense response genes^40, 41^. These, along with other candidate genes, suggest the importance of ET and JA signaling pathways in the defense against *S*. *sclerotiorum*.

#### Defense

Putative candidate genes with a defense functional category were involved in programmed plant cell death (PCD), both apoptosis and autophagy, or encoded genes related to pathogenesis-related (PR) genes and their products. Many genes in this category could also be dual-classified under signaling. Putative candidate gene *Glyma*.*12g169300*, associated with 14FLD, and *Glyma*.*12g171300*, associated with 15GHSE-DAI03, are homologous to barley (*Hordeum vulgare*) *MILDEW RESISTNACE LOCUS O* (*MLO*). A loss-of-function mutation in *mlo* genes confer resistance to powdery mildew fungus (*Erysiphe graminis*) in barley in a non-race-specific manner through the prevention of fungus penetration^42^. Additional mutations to the barley *MLO* locus show function in PCD and pathogen defense^43^.

*Glyma*.*13g328100*, associated with 15FLD, encodes a plant, small ubiquitin-like modifier (SUMO) E3 ligase homologous to *Arabidopsis SIZ1*, which has been shown to regulate innate immunity^44^. Mutant plants demonstrated elevated salicylic acid (SA) accumulation, expression of PR-genes, and increased resistance to *P*. *syringae*. Double mutants with genes such as *pad4* suggest the control of *SIZ1* on SA signaling. Mutations in *Arabidopsis pad4* gene reduce the expression of *PR-1* and levels of SA when infected with *P*. *syringae* ^45^. Together, *PAD4* and *SIZ1* work epistatically to regulate PR expression^44^. Finally, *Glyma*.*14g042400*, also associate with 15FLD, encodes a putative LRR-RLK transmembrane receptor induced by chitin oligomers homologous to Arabidopsis *RECETPOR LIKE PROTEIN 52* (*RLP52*). Chitin is a major component of fungal cell walls, and chitin oligomers are known to induce plant defense cascades.

### Transcript level of putative candidate genes

**Figure 2:**
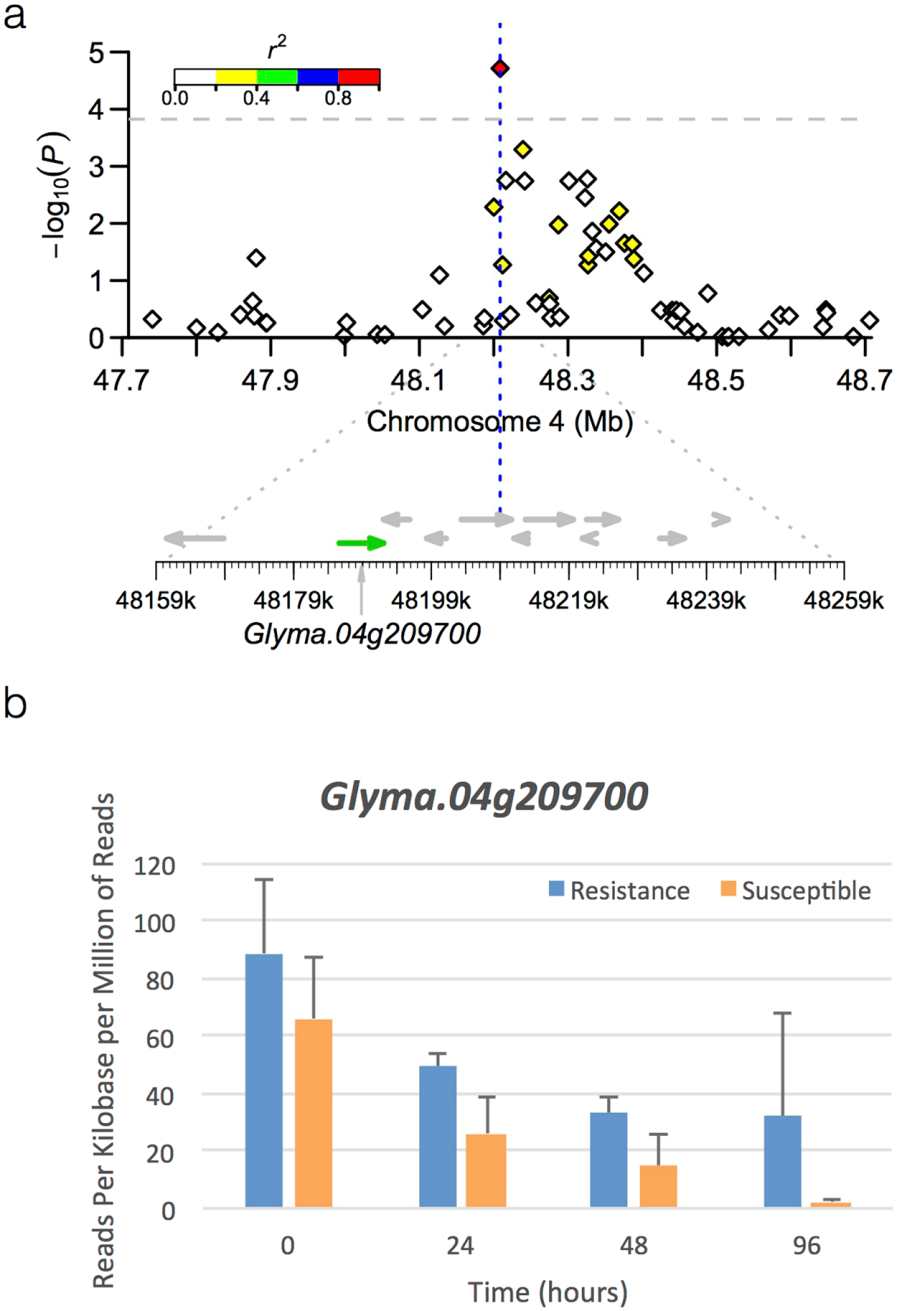
Regional association plot of *ss715588567* associated with 14GHSE-DAI03 and expression of the putative candidate gene *Glyma*.*04g209700*. (**a**) The top panel shows the negative log10-transformed *P* values from a genome-wide scan by using a mixed linear model (MLM) for plant severity taken 3 DAI within the adjacent region of peak SNP *ss715588567* plotted against base pair positions (Mb) on soybean chromosome 4. The significance threshold line is distinguished (gray dotted line), and each SNP depicts the extent of linkage disequilibrium in the region based on pairwise values with respect to the peak SNP. The values are indicated using the color intensity index shown. The bottom panel shows all putative genes in the region. The putative candidate gene indicated in green. (**b**) The expression of *Glyma*.*04g209700*, the putative candidate gene for *ss715588567*, in a modern resistant (blue) and susceptible (orange) cultivar. Error bars shown to indicate significance (p < 0.05).

**Figure 3:**
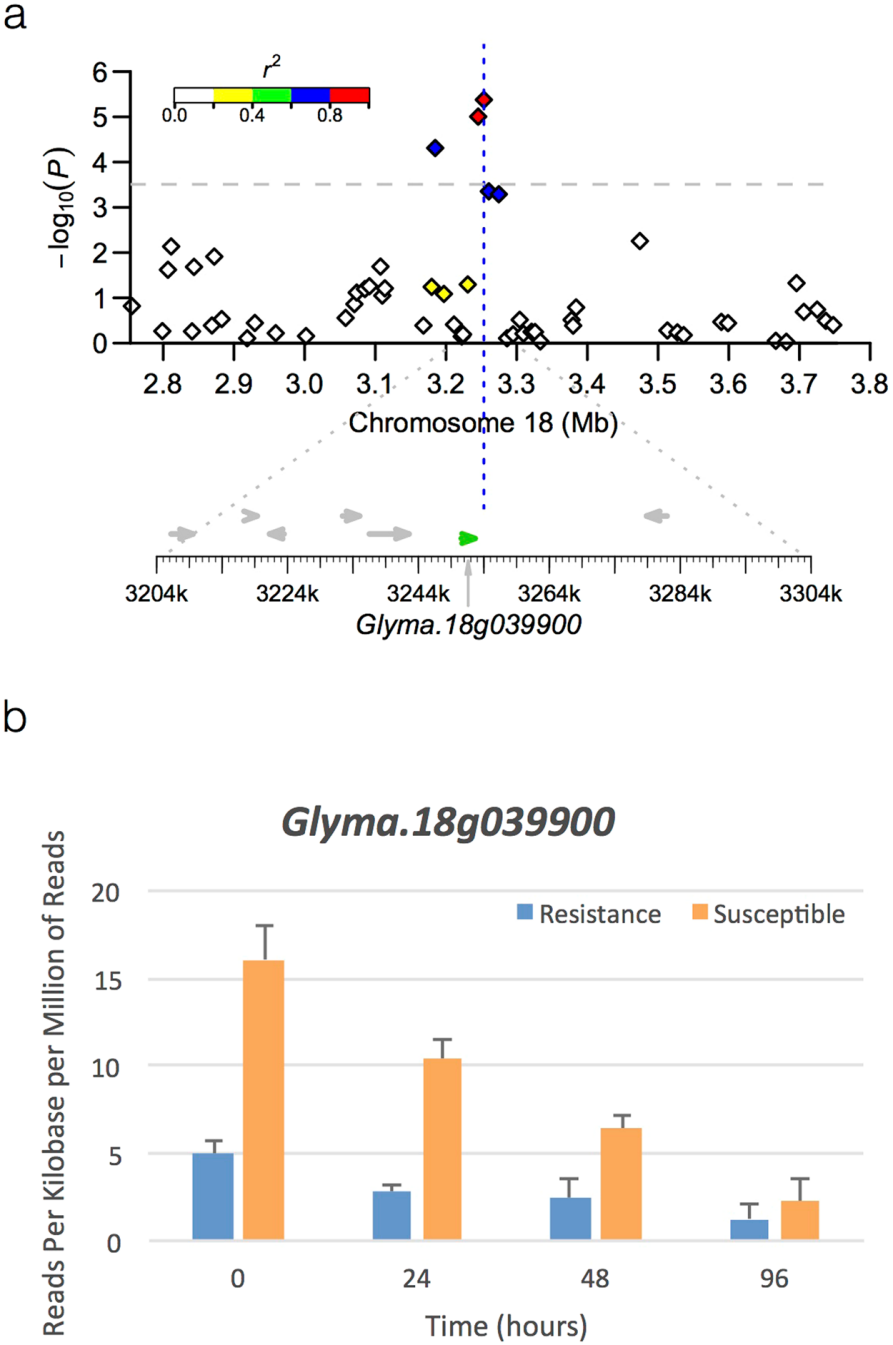
Regional association plot of *ss715630264* associated with 15GHSE-DAI03 and expression of the candidate gene *Glyma*.*18g039900*. (**a**) The top panel shows the negative log10-transformed *P* values from a genome-wide scan by using a mixed linear model (MLM) for plant severity taken 3 DAI within the adjacent region of peak SNP *ss715630264* plotted against base pair positions (Mb) on soybean chromosome 18. The significance threshold line is distinguished (gray dotted line), and each SNP depicts the extent of linkage disequilibrium in the region based on pairwise values with respect to the peak SNP. The values are indicated using the color intensity index shown. The bottom panel shows all putative genes in the region. The putative candidate gene indicated in green. (**b**) The expression of *Glyma*.*18g039900*, the putative candidate gene for *ss715630264*, in a modern resistant (blue) and susceptible (orange) cultivar. Error bars shown to indicate significance (p < 0.05).

An RNAseq dataset was mined for all available putative candidate genes detected through GWAS. This was done successfully for 38 putative candidate genes. Of these, 50% were differentially expressed between susceptible and resistant soybean lines. Out of the eight genes associated with DAI03, five were differentially regulated. *Glyma*.*04g209700*, a putative candidate gene (*PAD4*) associated with 14GHSE-DAI03, was consistently expressed at higher levels in the resistant line compared to the susceptible line throughout the time course of the experiment, and at significant levels at 24, 48, and 96 hours post-inoculation (Fig. 2). *Glyma*.*04g209700* encodes a lipase (class 3) protein and is homologous to *Arabidopsis PAD4*. *PAD4* is thought to be involved in the regulation of SA and has been shown to affect the activation of subsequent defense responses such as camalexin synthesis and *PR-1* gene expression^45, 46^. *Glyma*.*18g039900* was the putative candidate gene associated with 15GHSE-DAI03. Although the function of this gene is currently unknown, it was highly expressed in the susceptible line, and transcript levels were significantly higher compared to the resistant line (Fig. 3). Its association with SSR resistance in soybean makes this a gene of interest, and further studies addressing on its function are needed.

### GWES interaction analyses

Genome-wide epistatic interaction analysis had not yet been used in dissecting the genetic architecture of SSR resistance. Two loci SNP-SNP interactions were evaluated using a linear regression executed with PLINK^47, 48^. The number of significant interactions in field environments were much greater than those found significant in the greenhouse. This could be due to the additional environmental interactions present within these trials that are otherwise controlled by design within greenhouse trials. After clustering significant SNPs, a total of 22 and two significant SNP-SNP interactions were identified to be associated with 14FLD and 15FLD, respectively (Supplementary Table S4). The 37 unique loci involved within these interactions were reported on 13 different soybean chromosomes. Nine individual loci were involved in multiple interactions. No main effect SNPs had an additional significant epistatic interaction. Six of the 37 loci involved in significant SNP-SNP interactions co-localized with previously reported QTL for SSR resistance. When including all SNP-SNP interactions and additive effect SNPs, the explained phenotypic variation increased from 30–37% to 33–42% in field environments (Fig. 1).

Putative candidate genes were predicted for loci involved in SNP-SNP interactions. Genes annotated in SoyBase^12^ surrounding the peak SNP served as the source of potential candidate genes. A total of 26 candidate genes were predicted for loci involved in highly significant SNP-SNP interactions (Supplementary Table S4). One fifth of candidate genes were assigned to a membrane-related functional category, followed by signaling and cell wall functions. One category present that was not found among main effect loci was senescence.

#### Membrane

Genes involved in transport or gene products that are integral to the membrane were categorized as membrane-related. Candidate genes reported for highly significant epistatic loci contained transporters for both proteins and sugars. Sugars, including sucrose and its products, serve as important signaling molecules for multiple processes in a plant, including responses to biotic stresses^49, 50^. Pathogens may exploit plant sugar supplies which may subsequently trigger plant defense responses^50^. The expression of genes that may modify or reallocate sugar supply have been shown to be altered during these interactions^51, 52^. *Glyma*.*16g138800* encodes a vacuolar glucose exporter homologous to *Arabidopsis EARLY RESPONSIVE TO DEHDRATION-LIKE 6* (*AtERDL6*). This exporter responds to different stimuli; it is activated during darkness, heat, and wounding and repressed during cold stress^53, 54^. Since vacuoles are the major site for sugar storage, the activities of its sugar transporters can determine plant stress tolerance^55^. Interestingly, *Glyma*.*16g138800* is in an epistatic interaction with *Glyma*.*06g166800*, which encodes a sucrose efflux transporter homologous to *Arabidopsis SWEET12* (*AtSWEET12*) and *Medicago truncatula MTN3*. *Glyma*.*06g166800* is involved in several epistatic interactions with multiple other genes on Gm16 whose functions include sugar transporters, protein transporters, secondary metabolites, and transcription factors. *SWEET12* is localized in the plasma membrane of the phloem and mutants are hypothesized to limit sucrose availability in the apoplasm in order to prevent pathogen infection^56^.

#### Cell wall

Putative candidate genes with a cell wall functional category were involved in the synthesis or degradation of structural components such as cellulose and pectin. During infection, *S*. *sclerotiorum* facilitates penetration and degrades cell wall components by secreting a range of cell wall degrading enzymes (CWDEs)^3, 57, 58^. Therefore, cell wall composition is an important line of defense in host plants and has been shown to be differentially expressed between susceptible and resistant soybean genotypes infected with *S*. *sclerotiorum* ^37^. Putative candidate gene *Glyma*.*14g026200* encodes an acyl-esterase found in *powdery mildew resistant 5* (*pmr5*) and is homologous to *Arabidopsis TRICHOME BIREFRINGENCE-LIKE 27* (*TBL27*)^59, 60^. Arabidopsis plants containing a mutant *pmr5* gene were resistant to powdery mildew and contained cell walls that were pectin enriched with additional pectin modifications^59^.

#### Senescence

Two candidate genes specifically related to senescence were identified. These candidate genes had epistatic interactions with loci associated with signaling candidate genes that also play a role in flowering and senescence. Although senescence itself plays a minor role next to *S*. *sclerotiorum* oxalic acid-induced PCD^61^, these senescence genes may also function in the regulation of host PCD, thus directly affecting the establishment of *S*. *sclerotiorum* and disease outcome^62^. *Glyma*.*12g059200* encodes an alcohol dehydrogenase homologous to *Arabidopsis SENESCENCE-ASSOCIATED GENE 13* (*SAG13*). *SAG13* is an established marker gene for senescence. It lacks basal mRNA expression in young leaves and is detected in high amounts prior to the onset of senescence^63^. G*lyma*.*19g098500* encodes a glycerophosphoryl diester phosphodiesterase (GDPD) homologous to *Arabidopsis SENESCENCE-RELATED GENE 3* (*SRG3*) and *Arabidopsis GDPD1* ^64^.

Using a high mapping resolution, 58 main effect and 24 epistatic interactions were associated with SSR resistance in soybean when tested for various traits in multiple environments. Putative candidate genes identified multiple modes of resistance, including signaling pathways, defense-related, and cell wall functions establishing the complexity of resistance mechanisms. The lack of overlap in experiments is driven by differing mechanisms and loci, and point that plants have alternate systems to cope with SSR infection depending on the environmental conditions. Since *S*. *sclerotiorum* infects multiple different species, the results and discussion presented in this paper may be applicable to its many other hosts.

### Gene Networks

**Figure 4:**
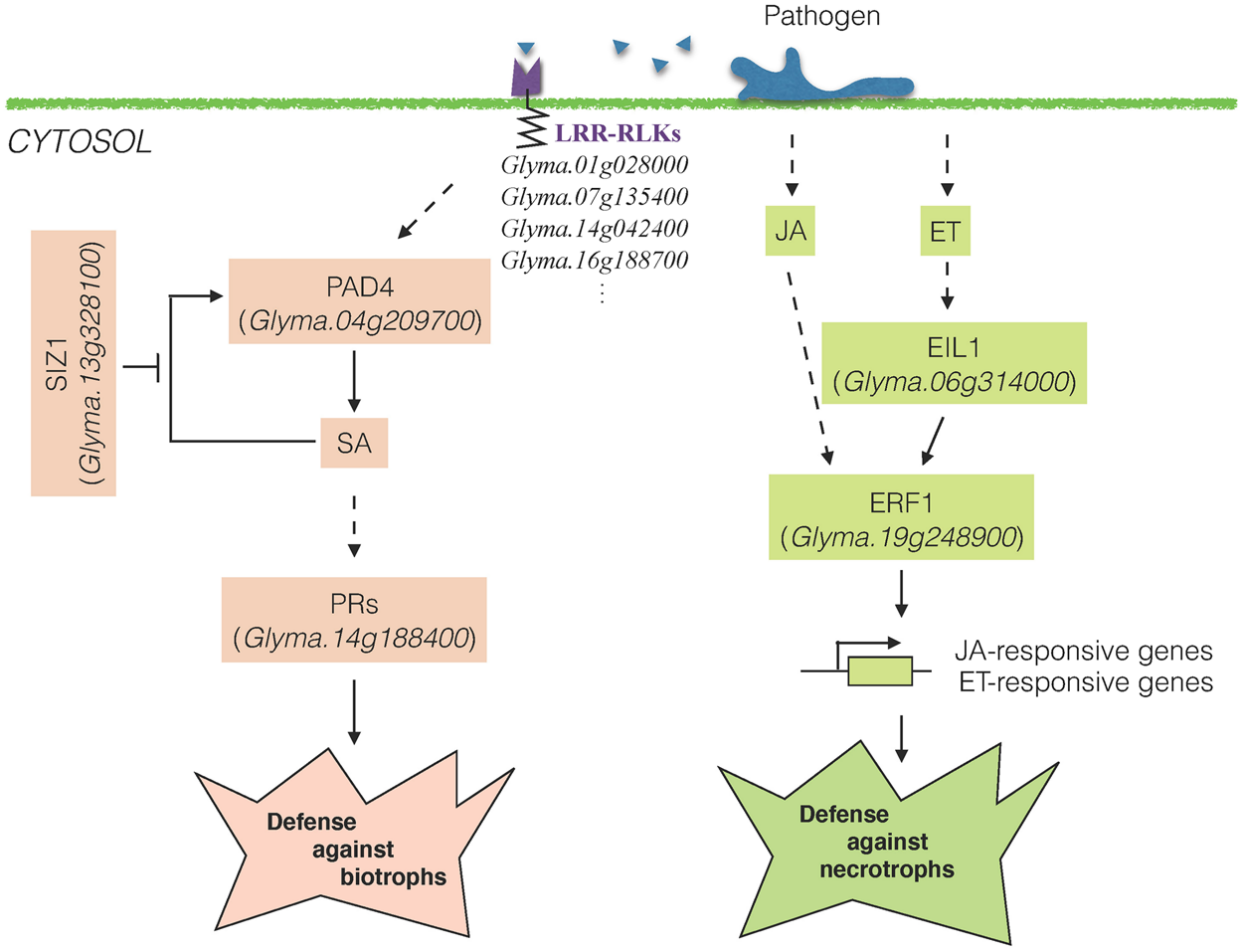
Proposed phytohormones-dependent disease defense model against *Sclerotinia sclerotiorum* in soybean. SA, salicylic acid; JA, jasmonic acid; ET, ethylene; LRR-RLKs, Leucine-rich repeat receptor-like protein kinases; PAD4, Phytoalexin-dependent4; SIZ1, small ubiquitin-related modifier E3 ligase; PRs, Pathogenesis-related proteins; EIL1, Ethylene-insensitive 3-like 1; ERF1, Ethylene response factor 1.

GWAS and GWES results were combined to propose a putative disease resistance model based on the *S*. *sclerotiorum* – soybean interaction, and the newly presumed hemibiotrophic nature of the fungus^65^. The putative candidate genes clustered into SA, JA, and ET pathways, indicating the possible involvement of a complex hormonal network typically activated by both necrotrophic (ET/JA) and biotrophic (SA) pathogens. This approach allowed the annotation of candidate genes with particular interest (*PAD4*, *EIL1*, and *ERF1*) identified in this study to the proposed model, giving support to the pathways of defense against biotrophs and necrotrophs for *S*. *sclerotiorum* (Fig. 4). The putative candidate genes, *PAD4*, *EIL1*, and *ERF1*, formed a gene network and included several other candidate genes identified in this study (Supplementary Fig. S7). Twenty-one putative candidate genes interacted at first level when *PAD4*, *ERF1*, and *EIL1* were considered (8 in SA, 9 in JA, and 5 in ET). The *PAD4* gene, as previously indicated, was significantly upregulated at 24, 48, and 96 hours in the RNAseq experiment, and seems to be crucial for the *S*. *sclerotiorum* – soybean disease interaction.

## Conclusions

Multiple resistant accessions identified in this study will be useful sources for the development of soybean cultivars with improved SSR resistance. Although greenhouse environments provide a less labor-intensive option for resistance screening, low correlations establish the importance of utilizing specialized SSR nurseries representative of field production systems. The complex genetic control of SSR resistance in soybean is evident with the identification of 58 main effect loci and 24 epistatic interactions. The reported candidate genes are involved in cell wall structure, hormone signaling and sugar allocation related to plant immunity, further revealing the complex nature of SSR resistance mechanisms. The results presented indicate that SA, JA, and ET pathways are important for resistance to SSR in soybean, and suggests that this pathogen-host response includes defenses against biotrophs and necrotrophs indicating a hemibiotrophic nature but requires functional validation to develop a more comprehensive model for *S*. *sclerotiorum*–soybean disease recognition and response pathway.

## Methods and Materials

### Plant material

Phenotypic evaluation consisted of 474 diverse soybean accessions from groups I, II, and III maturity as described by Hill *et al*.^66^. For genome-wide studies, genotypic information was available for 466 accessions. Accessions were obtained from the United States Department of Agriculture (USDA) Soybean Core Germplasm Collection curated by Dr. Randy Nelson in Urbana, IL^67^. DuPont Pioneer cultivars 92Y83 and 93M11 were grown as susceptible and resistant SSR performance checks, respectively. In 2015 field (15FLD) experiments, AxN-1-55 and PI567157A were grown as additional SSR resistant checks^68^.

### Phenotypic evaluation

#### Field environments

Soybean accessions were planted on May 23, 2014 and June 01, 2015 in a disease nursery field located at the Horticultural Research Station near Ames, IA with two replications in a randomized complete block (RCB) design. In both years, entries were planted in one-row, 1.5 m long plots, spaced 75 cm between adjacent plots. In 2014, plots were spaced with 1.5 m alleys and the seeding rate was five to six seeds per 30 cm. In 2015, alleys were reduced to 90 cm and the seeding rate was eight seeds per 30 cm to create a denser canopy and promote disease development and spread^9^.

*Sclerotinia sclerotiorum* culture used in 14FLD was started from surface-disinfested sclerotia collected in 2013 from a field in Northeast Iowa. After the 2014 growing season, sclerotia were collected from the SSR disease nursery field located near Ames, IA for use in the subsequent year. Cultures were maintained on potato dextrose agar (PDA) at room temperature. Soybean accessions were inoculated using the cotton pad method described by Bastien *et al*.^69^. The first set of inoculum was grown when maturity group I plots reached V5 growth stage^70^ on June 27, 2014 and July 08, 2015.

Inoculations took place at 7 am starting on July 03, 2014 and at 5 pm starting on July 15, 2015. Inoculations times were planned to avoid the hottest parts of the day to facilitate pathogen survival and infection. Plots that had reached crop growth stage R1^70^ (50% flowering) were inoculated by placing an infested cotton ball on the lowest flowering node on the main stem. Each plant in a plot was inoculated. There was a total of five inoculation days, spread six to seven days apart to ensure that each row was inoculated at the R1 growth stage. Fields were irrigated using overhead sprinklers until rating was completed to create an epiphytotic disease nursery.

Ratings were completed on Aug 26, 2014 and Sept 09, 2015 when plants reached the growth stage R5, about 30 days after inoculation (DAI). SSR disease ratings (per plant) were taken according to the system developed by Grau *et al*.^10^. The DS scale was based on a 0–3 scale: 0 = no symptoms, 1 = lateral branches showing lesions, 2 = lesions on main stem, and 3 = lesions on main stem resulting poor pod-fill or plant death.

#### Greenhouse environments

In 14GHSE, soybean accessions were evaluated using a RCB design with two replications. Entries were pre-germinated for 4–7 days at room temperature, and one healthy seedling was transplanted into a Ray Leach cone-tainer (Stuewe and Sons, Inc.) on Dec 15, 2014. Cones were placed on a rack with blank rows spaced between planted rows. Accessions were placed in a greenhouse set at 20–25 °C^26, 71^. Natural light was used in combination with high pressure sodium lights to provide a 16-hour photoperiod.

Soybean accessions were inoculated when the third trifoliate was fully expanded, approximately four weeks after transplanting. The inoculation procedure was adapted from Guo *et al*.^72^ and herein referred to as the cut-petiole method. *Sclerotinia sclerotiorum* cultures were started from surface-disinfested sclerotia collected in 2014 from SSR field experiment located near Ames, IA.

After inoculation, plants were placed inside a moisture chamber for a 48-hour incubation period. The chamber was constructed using PVC pipe and clear plastic. An Idylis Evaporative Humidifier was run at full capacity inside the moisture chamber to increase relative humidity during incubation. After the incubation period, the moisture chamber was removed and previous conditions were resumed.

In 15GHSE, phenotypic data was collected from a randomized layout with a single replication. Three seeds were directly planted into 8 oz. styrofoam cups for each soybean accession on Mar 31, 2015. At VC (unifoliate growth stage), cups were thinned down to two plants. Cups were spread out on April 23 and fertilized on April 29 with 200 ppm of Peters© Excel (15-5-15) to facilitate plant growth. Accessions were inoculated when the fifth trifoliate was fully expanded (V5)^70^, approximately six weeks after planting. Conditions and inoculation procedure were as described previously, cutting the third youngest fully expanded trifoliate.

Plants with SSR were rated for DS in both years. Disease severity was based on the scale previously described and taken on two separate dates: three and 14 DAI designated as DAI03 and DAI14, respectively. In 15GHSE experiments, WS and LL were measured. Wilt score was recorded as the number of days after inoculation that wilting or plant death was first observed. Scores were taken every other day from three to nine DAI, with a final rating at 14 DAI. Plants that did not observe wilting by the final rating were given a fixed score of 20 to represent resistance. Lesion length was recorded in mm as the length of visible lesion or discoloring on the main stem at seven DAI. This included lesions above and below the infection court.

### Statistical analysis of phenotypic data

All experiments and years were analyzed separately. When an experiment was replicated and the disease response was ordinal, an ordinal logistic regression with proportional odds assumption was used to obtain BLUPs for each accession (14FLD, 14GHSE-DAI03, 14GHSE-DAI14, and 15FLD). A mixed model approach was chosen to accommodate the unbalanced sample size among cultivars due to emergence rates. The analysis was performed using the *clmm* function (part of the ordinal package^73^) executed in the R statistical analysis software version 3.2.1^74^. The model for both field experiments (14FLD and 15FLD) was

$${Y}_{jklm}\sim Multinomial(1,{\pi }_{ijklm})$$

$$logit({\pi }_{ijklm})={\theta }_{i}+{R}_{j}+{A}_{k}+R{A}_{jk}+{D}_{l}+{S}_{m}+D{S}_{lm}$$

where *θ*_*i*_ is the intercept for the *i*th response category (*i* = 0, 1, 2), *R*_*j*_ is the effect of the *j*th replication, *A*_*k*_ is the effect of the *k*th accession, *RA*_*jk*_ is replication × accession interaction, *D*_*l*_ is the effect of the *l*th inoculation date, *S*_*m*_ is the effect of the *m*th range, and *DS*_*lm*_ is inoculation date × range interaction. The term *Y*_*jklm*_ represents a vector of ratings and *π*_*ijklm*_ is the probability that *Y*_*jklm*_ will be rated at or below the *i*th response category. Replication was assumed to be a fixed effect, and all other terms were assumed to be random effects. Similarly, the model for 14GHSE-DAI03 and 14GHSE-DAI14 was

$${Y}_{jk}\sim Multinomial(1,{\pi }_{ijk})$$

$${logit}({\pi }_{ijk})={\theta }_{i}+{R}_{j}+{A}_{k}.$$

Due to lack of replication in 15GHSE experiments, phenotypic data for each accession consisted of the average response of the two plants.

### Genotyping and quality control

The SNP data was prepared by Song *et al*.^27^ using the Illumina Infinium SoySNP50K BeadChip. For the soybean accessions involved in this study, data was downloaded from SoyBase^12^. Genotype data was accessible for 466 accessions and these accessions comprised the association mapping panel. Of the 42,180 SNPs available from these accessions, 284 SNPs that were presented in unanchored sequence scaffolds were excluded from further analyses. The dataset had a missing rate of 0.5%. Individual markers with missing rate larger than 10% were omitted and the remaining missing data were imputed using BEAGLE version 3.3.1 with default parameter settings^75, 76^. SNPs with a minor allele frequency (MAF) < 5% after imputation were also omitted for further analyses. A total 35,683 SNPs were used for GWAS and GWES.

Glyma.Wm.82.a2 reference genome was used to obtain chromosome physical lengths (bp) through SoyBase^12^ which were used to calculate genome-wide inter-marker distance and chromosome-wide densities. Pairwise LD between markers was measured using the squared correlation coefficient (*r* ^*2*^) between alleles using the R package synbreed^77^. Due to the variability of recombination between euchromatic and heterochromatic regions, *r* ^*2*^ was calculated separately for these two regions. SoyBase^12^ was used to outline the boundaries of the euchromatin and heterochromatin regions. The average LD decay presented used only the *r* ^*2*^ for SNPs with pairwise distance less than 10 Mb in either region of each chromosome by R script implementing the equation described in a previous study^78^. The LD decay rate of the population used was measured as the chromosomal distance where the average *r* ^*2*^ dropped to half of its maximum value^79^.

Nucleotide diversity was measured by polymorphism information content (PIC). The equation used was

$$PI{C}_{i}=1-(MA{F}_{i}^{2}+{(1-MA{F}_{i})}^{2})-2* MA{F}_{i}^{2}* {(1-MA{F}_{i})}^{2},$$

where *MAF*_*i*_ is the minor allele frequency of the *i*th SNP^80^. The average PIC was calculated at 100 kb intervals.

### Genome-wide association (GWAS) and epistatic (GWES) analyses

Phenotypic data of each rating for individual accessions were used to fit the one-way ANOVA model for naïve test (without correction for familial relatedness or population structure) implemented in R^74^ and three types of models, general linear model (GLM), mixed linear model (MLM), and compressed MLM (cMLM), implemented in the GAPIT software for marker-trait association analyses^81, 82^. The threshold for significant associations was determined by the empirical significance level of *P* < 0.001. This was evaluated by performing 1,000 permutations of the GWAS as previously described^16^. In a genotypic file where a row represented an individual accession and a column represented a single SNP, rows were randomly shuffled without changing the row names. This was repeated for each iteration. The threshold was set as the lowest *P* value of the SNP-trait association that did not meet the empirical significance level.

Genome-wide epistatic interactions test were carried out in PLINK^47, 48^. The equation implemented was

$$y={b}_{0}+{b}_{1}A+{b}_{2}B+{b}_{3}AB+e,$$

where *b*_0_ is the overall mean, *b*_1_ is the additive effects of marker *A*, *b*_2_ is the additive effects of marker *B*, *b*_3_ is the interaction effect between markers *A* and *B*, and *e* is the random error following $N(0,{\sigma }_{e}^{2})$. Due to the high number of significant interaction, a more stringent Bonferroni threshold (*α* = 1 × 10^−10^) was used to eliminate small effect interactions that would give a minimal impact on trait expression and genetic enhancement applications for field resistance to SSR.

The proportion of phenotypic variance (*R* ^2^) explained by significant peak main effect SNPs or the significant peak main effect SNPs plus significant epistatic effect SNP-SNP interactions were calculated for each trait using a fixed linear regression model.

### Prediction of candidate genes

Genes annotated in Glyma1.1, Glmy1.0, and NCBI RefSeq gene models available through SoyBase aligning to the Glyma.Wm.82.a2 reference genome^12^ were used as the source of candidate genes. A 50 kB region on either side of peak SNPs were investigated unless significant SNPs were involved in a linkage block with *r* ^*2*^ > 0.7 in respect to the peak SNP. At this point, the more stringent method was used, resulting in a smaller region to investigate. The peak SNP is defined as the SNP with the lowest *P* value. The prediction of candidate genes resulted from the following priorities: (i) genes of known function in soybean related to disease resistance, (ii) genes of function-known orthologs in *Arabidopsis* related to disease resistance, and (iii) genes pinpointed by the peak SNPs. Functional categories were assigned to each putative candidate gene using the criteria outlined by Calla *et al*.^37^ based on information given by annotations available in SoyBase^12^ and TAIR^83^.

### Next generation RNA sequencing

Two isogenic breeding lines of soybean (*Glycine max*); susceptible (91–44) and partially resistant (91–145) were used for the experiment. Four week old soybean plants were infected with *S*. *sclerotiorum* by petiole inoculation method^11^ and stem tissue was collected at 0 (control), 24, 48, and 96 hours post inoculation. The experimental design consisted of three biological replicates for each of the treatments. Total RNA was extracted from the plant tissues (stem segment) following the standard Trizol protocol (Invitrogen Corp., Carlsbad, CA, USA), further cleaned up using RNeasy plant mini kit protocol (RNeasy Plant Mini Kit, Qiagen, cat. no. 74904, Hilden, Germany). RNA from each sample was randomly fragmented, and individually indexed libraries were prepared using the TruSeq RNA Sample Preparation v2 kit according to the manufacturer’s instructions (Illumina, San Diego CA, USA). After additional processing, cDNA was sequenced using Illumina HiSeq2000 (Illumina, San Diego CA, USA). Low-quality bases (Q < 20) were trimmed out from both the 5′ and 3′ ends of the reads. The quality of each library was controlled with FastQC. Resulting reads were aligned to the reference soybean genome Wm82.a2.v1^12^ using R Limma package, bioconductor release version (3.3)^84^. Data was normalized and differential gene expression was calculated for each gene in reads per kilobase per million (RPKM).

### Identification of *Arabidopsis* genes corresponding to soybean (*Glyma*) genes and Gene network analyses

*Arabidopsis* (TAIR) genes and the corresponding Glyma (soybean) genes were identified using Tools option with gene annotation lookup at www.soybase.org. These TAIR genes were used in Cytoscape 3.4.0 version with option of Genemania (load data) network of 315 considering 27,416 TAIR genes of 142,800,814 interactions^85^. The interactome was obtained with default settings for all putative candidate genes found in this study.

## Electronic supplementary material

Supplementary Info

## References

[1] Boland GJ, Hall R (1994). Index of plant hosts of Sclerotinia sclerotiorum. Can. J. Plant Pathol..

[2] Purdy LH (1979). *Sclerotinia sclerotiorum*: History, Diseases and Symptomatology, Host Range, Geographic Distribution, and Impact. Phytopathology.

[3] Bolton MD, Thomma BPHJ, Nelson BD (2006). Sclerotinia sclerotiorum (Lib.) de Bary: biology and molecular traits of a cosmopolitan pathogen. Mol. Plant Pathol..

[4] Grau, C. R. & Hartman, G. L. Sclerotinia stem rot. Compend. Soybean Dis. 4th Ed APS Press St Paul MN Sclerotinia Stem Rot 46–48 (1999).

[5] Koenning, S. R. & Wrather, J. A. Suppression of Soybean Yield Potential in the Continental United States by Plant Diseases from 2006 to 2009. *Plant Health Prog*. **Online**, (2010).

[6] Bradley, C., Allen, T., Esker, P., Wrather, J. A. & Koenning, S. Estimates of soybean yield reductions caused by diseases in the United States.

[7] Hoffman DD (1998). Yield and Seed Quality of Soybean Cultivars Infected with Sclerotinia sclerotiorum. Plant Dis..

[8] Mueller DS (2002). Efficacy of Fungicides on Sclerotinia sclerotiorum and Their Potential for Control of Sclerotinia Stem Rot on Soybean. Plant Dis..

[9] Peltier AJ (2012). Biology, Yield loss and Control of Sclerotinia Stem Rot of Soybean. J. Integr. Pest Manag..

[10] Grau CR, Radke VL, Gillespie FL (1982). Resistance of Soybean Cultivars to Sclerotinia sclerotiorum. Plant Dis..

[11] Hoffman DD (2002). Selected Soybean Plant Introductions with Partial Resistance to Sclerotinia sclerotiorum. Plant Dis..

[12] Grant D, Nelson RT, Cannon SB, Shoemaker RC (2010). SoyBase, the USDA-ARS soybean genetics and genomics database. Nucleic Acids Res..

[13] Hwang E-Y (2014). A genome-wide association study of seed protein and oil content in soybean. BMC Genomics.

[14] Mamidi S, Lee RK, Goos JR, McClean PE (2014). Genome-Wide Association Studies Identifies Seven Major Regions Responsible for Iron Deficiency Chlorosis in Soybean (Glycine max). PLoS ONE.

[15] Sonah H, O’Donoughue L, Cober E, Rajcan I, Belzile F (2015). Identification of loci governing eight agronomic traits using a GBS-GWAS approach and validation by QTL mapping in soya bean. Plant Biotechnol. J..

[16] Zhang J (2015). Genome-wide association study for flowering time, maturity dates and plant height in early maturing soybean (Glycine max) germplasm. BMC Genomics.

[17] Bastien M, Sonah H, Belzile F (2014). Genome Wide Association Mapping of Sclerotinia sclerotiorum Resistance in Soybean with a Genotyping-by-Sequencing Approach. Plant Genome.

[18] Iquira E, Humira S, François B (2015). Association mapping of QTLs for sclerotinia stem rot resistance in a collection of soybean plant introductions using a genotyping by sequencing (GBS) approach. BMC Plant Biol..

[19] Zhao X (2015). Loci and candidate gene identification for resistance to *Sclerotinia sclerotiorum* in soybean (*Glycine max* L. Merr.) via association and linkage maps. Plant J..

[20] Wegulo SN, Yang XB, Martinson CA (1998). Soybean Cultivar Responses to Sclerotinia sclerotiorum in Field and Controlled Environment Studies. Plant Dis..

[21] Kim HS (2000). Reaction of Soybean Cultivars to Sclerotinia Stem Rot in Field, Greenhouse, and Laboratory Evaluations. Crop Sci..

[22] Manolio TA (2009). Finding the missing heritability of complex diseases. Nature.

[23] Zhang J, Singh A, Mueller DS, Singh AK (2015). Genome-wide association and epistasis studies unravel the genetic architecture of sudden death syndrome resistance in soybean. Plant J..

[24] Boland GJ, Hall R (1987). Evaluating Soybean Cultivars for Resistance to Sclerotinia sclerotiorum Under Field Conditions. Plant Dis..

[25] Chun D, Kao LB, Lockwood JL, Isleib TG (1987). Laboratory and Field Assessment of Resistance in Soybean to Stem Rot Caused by Sclerotinia sclerotiorum. Plant Dis..

[26] Nelson BD, Helms TC, Olson MA (1991). Comparison of Laboratory and Field Evaluations of Resistance in Soybean to *Sclerotinia sclerotiorum*. Plant Dis..

[27] Song Q (2013). Development and Evaluation of SoySNP50K, a High-Density Genotyping Array for Soybean. PLoS ONE.

[28] Schmutz J (2010). Genome sequence of the palaeopolyploid soybean. Nature.

[29] Rafalski JA (2010). Association genetics in crop improvement. Curr. Opin. Plant Biol..

[30] Lam H-M (2010). Resequencing of 31 wild and cultivated soybean genomes identifies patterns of genetic diversity and selection. Nat. Genet..

[31] Wen Z (2014). Genome-wide association mapping of quantitative resistance to sudden death syndrome in soybean. BMC Genomics.

[32] Li Y-H (2010). Genetic diversity in domesticated soybean (Glycine max) and its wild progenitor (Glycine soja) for simple sequence repeat and single-nucleotide polymorphism loci. New Phytol..

[33] Arahana VS, Graef GL, Specht JE, Steadman JR, Eskridge KM (2001). Identification of QTLs for Sclerotinia sclerotiorum Resistance to in Soybean. Crop Sci..

[34] Malmberg RL, Held S, Waits A, Mauricio R (2005). Epistasis for Fitness-Related Quantitative Traits in Arabidopsis thaliana Grown in the Field and in the Greenhouse. Genetics.

[35] Messmer R (2009). Drought stress and tropical maize: QTL-by-environment interactions and stability of QTLs across environments for yield components and secondary traits. Theor. Appl. Genet..

[36] Li, P. *et al*. Use of genotype-environment interactions to elucidate the pattern of maize root plasticity to nitrogen deficiency: Root plasticity to nitrogen in maize. *J*. *Integr*. *Plant Biol*. 1–12, doi:10.1111/jipb.12384 (2015). 10.1111/jipb.12384 26269087

[37] Calla B, Vuong T, Radwan O, Hartman GL, Clough SJ (2009). Gene Expression Profiling Soybean Stem Tissue Early Response to Sclerotinia sclerotiorum and In Silico Mapping in Relation to Resistance Markers. Plant Genome J..

[38] Gururani MA (2012). Plant disease resistance genes: Current status and future directions. Physiol. Mol. Plant Pathol..

[39] Zhu Z (2011). Derepression of ethylene-stabilized transcription factors (EIN3/EIL1) mediates jasmonate and ethylene signaling synergy in Arabidopsis. Proc. Natl. Acad. Sci..

[40] Lorenzo O, Piqueras R, Sánchez-Serrano JJ, Solano R (2003). ETHYLENE RESPONSE FACTOR1 Integrates Signals from Ethylene and Jasmonate Pathways in Plant Defense. Plant Cell.

[41] Berrocal-Lobo M, Molina A, Solano R (2002). Constitutive expression of ETHYLENE-RESPONSE-FACTOR1 in Arabidopsis confers resistance to several necrotrophic fungi. Plant J..

[42] Jørgensen IH (1992). Discovery, characterization and exploitation of Mlo powdery mildew resistance in barley. Euphytica.

[43] Devoto A (1999). Topology, subcellular localization, and sequence diversity of the Mlo family in plants. J. Biol. Chem..

[44] Lee J (2007). Salicylic acid-mediated innate immunity in Arabidopsis is regulated by SIZ1 SUMO E3 ligase. Plant J..

[45] Zhou N, Tootle TL, Tsui F, Klessig DF, Glazebrook J (1998). PAD4 Functions Upstream from Salicylic Acid to Control Defense Responses in Arabidopsis. Plant Cell.

[46] Jirage D (1999). Arabidopsis thaliana PAD4 encodes a lipase-like gene that is important for salicylic acid signaling. Proc. Natl. Acad. Sci. USA..

[47] Purcell S (2007). PLINK: A Tool Set for Whole-Genome Association and Population-Based Linkage Analyses. Am. J. Hum. Genet..

[48] Purcell, S. *PLINK v1*.*07*.

[49] Rolland F, Moore B, Sheen J (2002). Sugar Sensing and Signaling in Plants. Plant Cell.

[50] Tauzin AS, Giardina T (2014). Sucrose and invertases, a part of the plant defense response to the biotic stresses. Front. Plant Sci..

[51] Sutton PN, Gilbert MJ, Williams LE, Hall JL (2007). Powdery mildew infection of wheat leaves changes host solute transport and invertase activity. Physiol. Plant..

[52] Hyun TK, Eom SH, Rim Y, Kim J-S (2011). Alteration of the expression and activation of tomato invertases during Botrytis cinerea infection. Plant Omics.

[53] Poschet G (2011). A novel Arabidopsis vacuolar glucose exporter is involved in cellular sugar homeostasis and affects the composition of seed storage compounds. Plant Physiol..

[54] Klemens PAW (2014). Overexpression of a proton-coupled vacuolar glucose exporter impairs freezing tolerance and seed germination. New Phytol..

[55] Hedrich R, Sauer N, Neuhaus HE (2015). Sugar transport across the plant vacuolar membrane: nature and regulation of carrier proteins. Curr. Opin. Plant Biol..

[56] Chen L-Q (2012). Sucrose Efflux Mediated by SWEET Proteins as a Key Step for Phloem. Transport. Science.

[57] Riou C, Freyssinet G, Fevre M (1991). Production of Cell Wall-Degrading Enzymes by the Phytopathogenic Fungus Sclerotinia sclerotiorum. Appl. Environ. Microbiol..

[58] Lumsden RD (1979). History and physiology of pathogenesis in plant diseases caused by Sclerotinia species. Phytopathology.

[59] Vogel JP, Raab TK, Somerville CR, Somerville SC (2004). Mutations in PMR5 result in powdery mildew resistance and altered cell wall composition. Plant J..

[60] XiaoFang, Z. *et al*. TBL27 Affects Aluminium Sensitivity by Modulating the O-acetylation of Xyloglucan and Aluminium Binding Capacity in Arabidopsis. *Plant Physiol*. pp.114.243808, doi:10.1104/pp.114.243808 (2014). 10.1104/pp.114.243808 PMC4149705 25006026

[61] Kim KS, Min J-Y, Dickman MB (2008). Oxalic Acid Is an Elicitor of Plant Programmed Cell Death during Sclerotinia sclerotiorum Disease Development. Mol. Plant. Microbe Interact..

[62] Kabbage M, Williams B, Dickman MB (2013). Cell Death Control: The Interplay of Apoptosis and Autophagy in the Pathogenicity of Sclerotinia sclerotiorum. PLoS Pathog..

[63] Weaver LM, Gan S, Quirino B, Amasino RM (1998). A comparison of the expression patterns of several senescence-associated genes in response to stress and hormone treatment. Plant Mol. Biol..

[64] Cheng Y (2011). Characterization of the Arabidopsis glycerophosphodiester phosphodiesterase (GDPD) family reveals a role of the plastid-localized AtGDPD1 in maintaining cellular phosphate homeostasis under phosphate starvation. Plant J..

[65] Kabbage M, Yarden O, Dickman MB (2015). Pathogenic attributes of Sclerotinia sclerotiorum: Switching from a biotrophic to necrotrophic lifestyle. Plant Sci..

[66] Hill, J. H. *et al*. Evaluation of the USDA Soybean Germplasm Collection: Maturity Groups 000 to IV (PI 507670 to PI 574486). *US Dep*. *Agric*. *Tech*. *Bull*. *No 1914* (2005).

[67] Oliveira MF, Nelson RL, Geraldi IO, Cruz CD, de Toledo JFF (2010). Establishing a soybean germplasm core collection. Field Crops Res..

[68] Diers BW (2006). Registration of AxN-1-55 soybean germplasm with partial resistance to Sclerotinia stem rot. Crop Sci..

[69] Bastien M (2012). A reproducible assay for measuring partial resistance to Sclerotinia sclerotiorum in soybean. Can. J. Plant Sci..

[70] Fehr WR, Caviness CE, Burmood DT, Pennington JS (1971). Stage of Development Descriptions for Soybeans, Glycine Max (L.) Merrill1. Crop Sci..

[71] Abawi GS, Grogan RG (1975). Source of Primary Inoculum and Effects of Temperature and Moisture on Infection of Beans by Whetzelinia sclerotiorum. Phytopathology.

[72] Guo X (2008). Genetic Mapping of QTLs Underlying Partial Resistance to in Soybean PI 391589A and PI 391589B. Crop Sci.

[73] Christensen, R. H. B. *ordinal: Regression Models for Ordinal Data* (2015).

[74] R Core Team. *R: A Language and Environment for Statistical Computing*. (R Foundation for Statistical Computing, 2015).

[75] Browning BL, Browning SR (2007). Efficient multilocus association testing for whole genome association studies using localized haplotype clustering. Genet. Epidemiol..

[76] Browning BL, Browning SR (2009). A Unified Approach to Genotype Imputation and Haplotype-Phase Inference for Large Data Sets of Trios and Unrelated Individuals. Am. J. Hum. Genet..

[77] Wimmer V, Albrecht T, Auinger H, Schoen C (2012). synbreed: a framework for the analysis of geomic prediction data using R. Bioinformatics.

[78] Remington DL (2001). Structure of linkage disequilibrium and phenotypic associations in the maize genome. Proc. Natl. Acad. Sci..

[79] Huang X (2010). Genome-wide association studies of 14 agronomic traits in rice landraces. Nat. Genet..

[80] Nagy S (2012). PICcalc: An Online Program to Calculate Polymorphic Information Content for Molecular Genetic Studies. Biochem. Genet..

[81] Zhang Z (2010). Mixed linear model approach adapted for genome-wide association studies. Nat. Genet..

[82] Lipka AE (2012). GAPIT: genome association and prediction integrated tool. Bioinformatics.

[83] Lamesch P (2012). The Arabidopsis Information Resource (TAIR): improved gene annotation and new tools. Nucleic Acids Res..

[84] Ritchie ME (2015). limma powers differential expression analyses for RNA-sequencing and microarray studies. Nucleic Acids Res..

[85] Shannon P (2003). Cytoscape: a software environment for integrated models of biomolecular interaction networks. Genome Res..

