# Supplementary material for: Main and epistatic loci studies in soybean for Sclerotinia sclerotiorum resistance reveal multiple modes of resistance in multi-environments
Source: Sci Rep. 2017 Jun 15;7:3554. doi: 10.1038/s41598-017-03695-9 (PMC5472596; doi:10.1038/s41598-017-03695-9)
Supplement: Supplementary file 1 — Supplementary Info [file 41598_2017_3695_MOESM1_ESM.pdf]

Main and epistatic loci studies in soybean for *Sclerotinia sclerotiorum* resistance reveal multiple modes of resistance in multi-environments

Tara C. Moellers[1], Arti Singh[1], Jiaoping Zhang[1], Jae Brungardt[1], Mehdi Kabbage[2], Daren S. Mueller[3], Craig R. Grau[2], Ashish Ranjan[2], Damon L. Smith[2], RV Chowda-Reddy[1], Asheesh K. Singh[1*]

[1] Department of Agronomy, Iowa State University, Ames, Iowa, 50011, United States of America

[2] Department of Plant Pathology, University of Wisconsin-Madison, Madison, Wisconsin, 53706, United States of America

[3] Department of Plant Pathology, Iowa State University, Ames, Iowa, 50011, United States of America

[*] singhak@iastate.edu

Supplementary Information

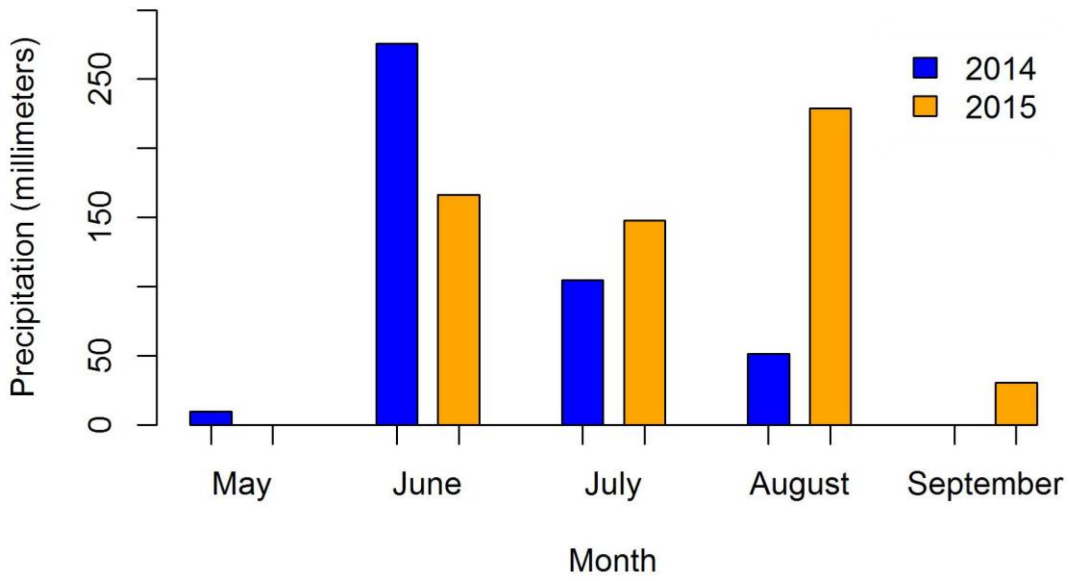

Figure S1. Precipitation over growing season at a disease nursery near Ames, IA in 2014 and 2015. Measurements taken from the day of planting until the last day of rating in millimeters. Data from 2014 shown in blue and 2015 in gold.

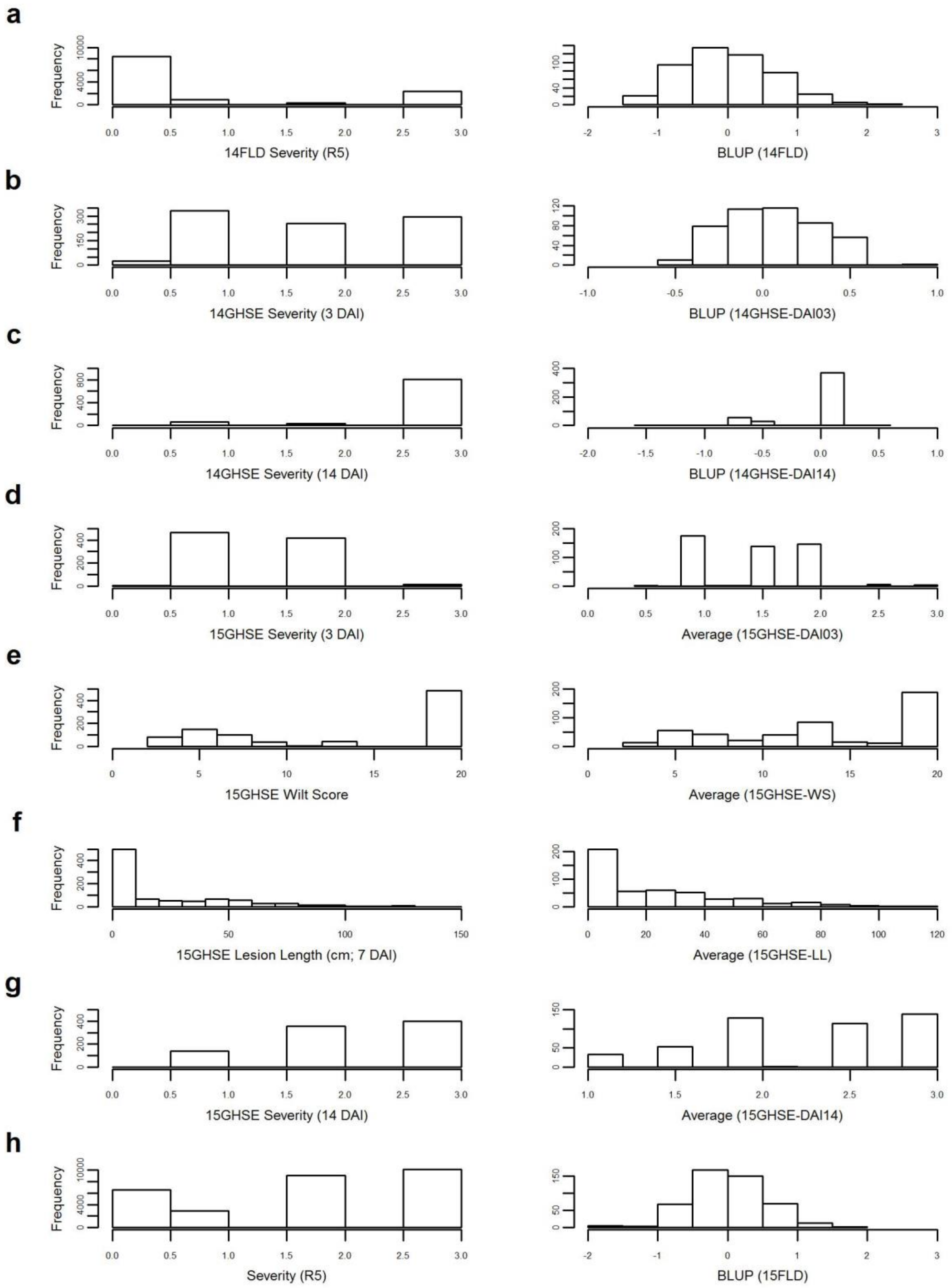

Figure S2. Distribution of Sclerotinia stem rot response ratings before regression and distribution of genotypic values after regression.

Distribution of ratings in the soybean panel are shown in the right column, distribution of genotypic values shown in left, for (a)14FLD Severity, (b) 14GHSE-DAI03, (c) 14GHSE-DAI14, (d) 15GHSE-DAI03, (e) 15GHSE-WS, (f) 15GHSE-LL, (g) 15GHSE-DAI14, and (h) 15FLD Severity.

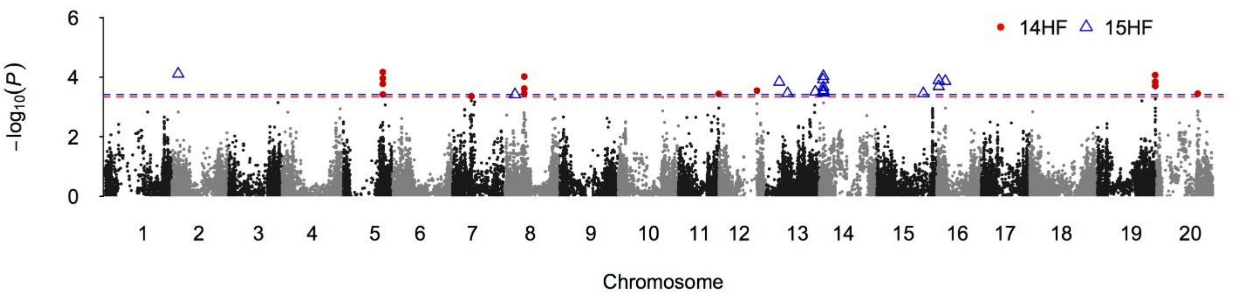

Figure S3. Manhattan plots of genome-wide association study for plant severity in field environments. Overlaid negative log10-transformed *P* values from a genome-wide scans using a mixed linear model (MLM) for 2014 field trail (14FLD) and compressed MLM for 2015 field trail (15FLD) plotted against base pair positions of each SNP on each of the 20 soybean chromosomes. The significance threshold line is distinguished (dotted line) for 2014 (red) and 2015 (blue). Significant trait-associated SNPs are highlighted for 2014 (red dot) and 2015 (blue triangle).

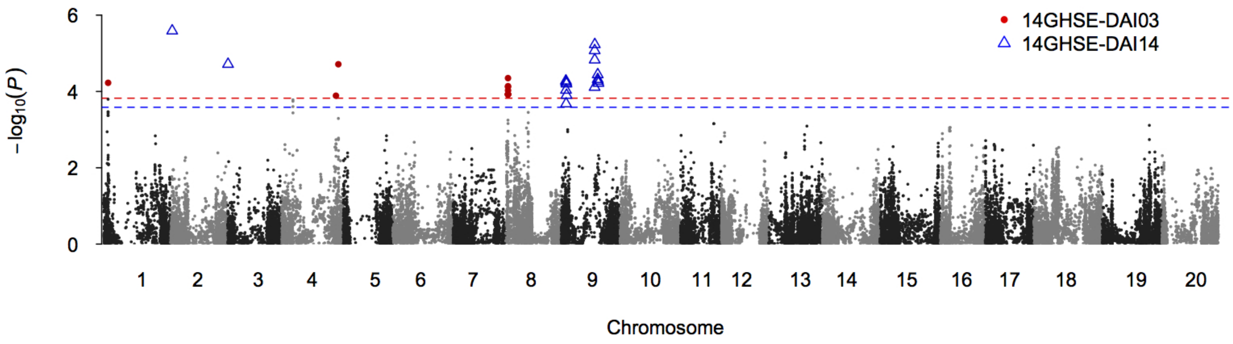

Figure S4. Manhattan plots of genome-wide association study for traits measured in 2014 greenhouse environments. Overlaid negative log10-transformed *P* values from genome-wide scans using a mixed linear model (MLM) for plant severity taken 3 DAI (DAI03) and 14 DAI (DAI14) plotted against base pair positions of each SNP on each of the 20 soybean chromosomes. The significance threshold line is distinguished (dotted line) for DAI03 (red) and DAI14 (blue). Significant trait-associated SNPs are highlighted for DAI03 (red dot) and DAI14 (blue triangle).

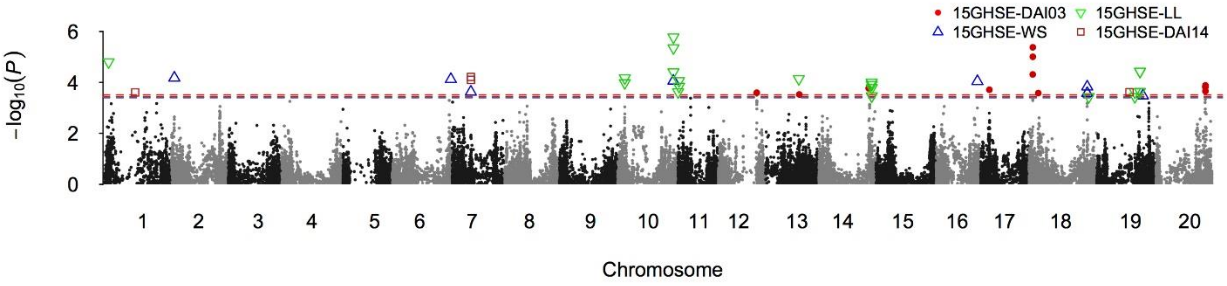

Figure S5. Manhattan plots of genome-wide association study for traits measured in 2015 greenhouse environments. Overlaid negative log10-transformed P values from a genome-wide scan by using a mixed linear model (MLM) for plant severity taken 3 DAI (DAI03), wilt score (WS), and plant severity taken 14 DAI (DAI14) and compressed MLM for lesion length (LL) plotted against base pair positions of each SNP on each of the 20 soybean chromosomes. The significance threshold line is distinguished for DAI03 (red), WS (blue), LL (green), and DAI14 (maroon), and all significant trait-associated SNPs are highlighted for DAI03 (red dot), WS (blue triangle), LL (green nabla) and DAI14 (maroon square).

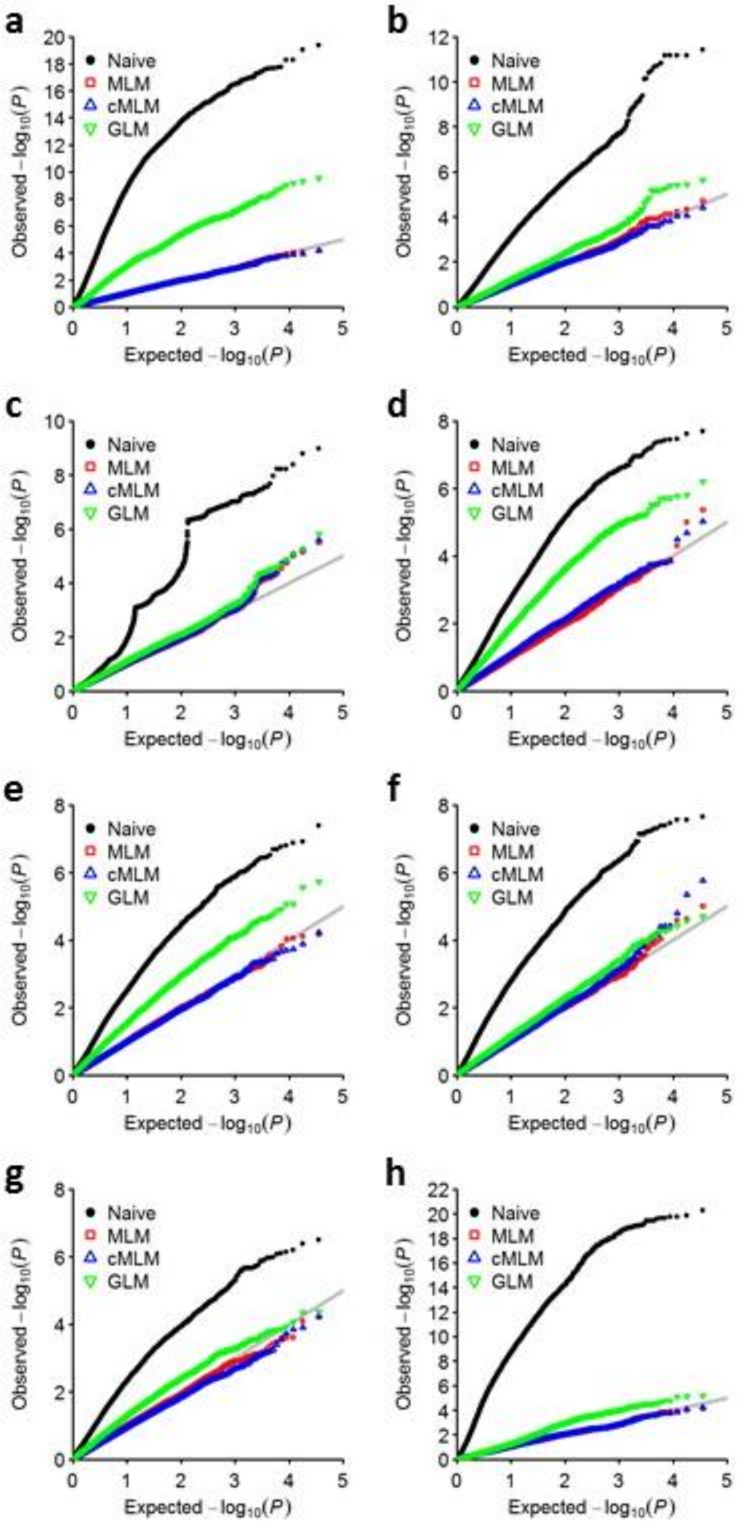

Figure S6: Quantile-quantile plots of the genome-wide association results for Sclerotinia stem rot resistance in a diverse soybean panel shown for the naïve model and linear models for each trait measured. The naive model (black), GLM (green), MLM (red), and cMLM (blue)

are presented for (a)14FLD Severity, (b) 14GHSE-DAI03, (c) 14GHSE-DAI14, (d) 15GHSE-DAI03, (e) 15GHSE-WS, (f) 15GHSE-LL, (g) 15GHSE-DAI14, and (h) 15FLD Severity. The expected distribution of negative log_{10}-transformed *P* values is indicated in gray.

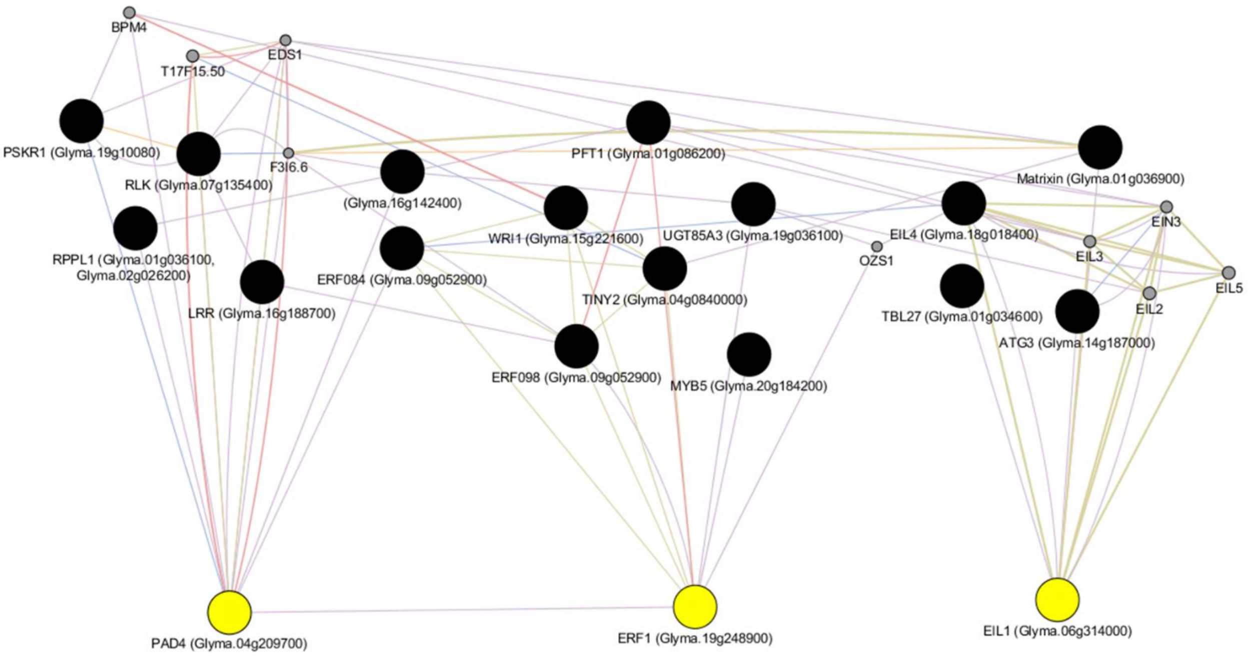

Figure S7: First level interact genes identified in this study involved in Salicylic Acid, Jasmonic Acid and Ethylene pathways. The genes provided in Glyma format are genes identified in this study and others showed in TAIR (Arabidopsis) format that form an interactome. Different color lines represent interactions by co-expression, physical, shared protein domains, predicated and co-localization studies (refer Shannon et al. 2003).

Table S1. Spearman coefficients of rank correlation between different experiments and measurements of Sclerotinia stem rot disease resistance from the soybean association panel.

	14FLD	14 GHSE		15 GHSE				15FLD
**Trait**	**Severity**	**DAI03**	**DAI14**	**DAI03**	**WS**	**LL**	**DAI14**	**Severity**
**14FLD**								
Severity	1.00	…	…	…	…	…	…	…
**14GHSE**								
DAI03	0.15**	1.00	…	…	…	…	…	…
DAI14	-0.02	0.46***	1.00	…	…	…	…	…
**15GHSE**								
DAI03	0.17***	0.04	-0.02	1.00	…	…	…	…
WS	-0.14**	-0.09	-0.04	-0.63***	1.00	…	…	…
LL	0.17***	0.10*	0.03	0.68***	-0.90***	1.00	…	…
DAI14	0.13**	0.09	0.02	0.63***	-0.90***	0.84***	1.00	…
**15FLD**								
Severity	0.32***	0.12**	-0.01	0.2***	-0.15**	0.2***	0.17***	1.00

14FLD = 2014 field, 14GHSE = 2014 greenhouse, 15GHSE= 2015 greenhouse, and 15FLD=2015 field environments.

DAI03, DAI14 = Plant severity score given at 3 and 14 DAI, WS = wilt score, and LL = lesion length.

*,**,***=Significant at the 0.05, 0.01, and 0.001 probability levels.

Table S2. Genotypic values of disease checks and designated accessions from the diverse soybean panel phenotyped for Sclerotinia stem rot resistance in multiple greenhouse and field environments for multiple traits.

Line	14FLD	14GHSE		15GHSE				15FLD
	Severity	DAI03	DAI14	DAI03	WS	LL	DAI14	Severity
**Accessions**								
Lowest Disease Rating	-1.49	-0.56	-1.42	0.50	20.00	1.00	1.00	-1.84
Highest Disease Rating	2.24	2.24	0.13	3.00	3.00	119.50	3.00	1.64
**Disease check cultivars**								
92Y83 (Susceptible)	0.58	0.98	0.46	1.30	15.70	27.40	2.10	0.57
93M11 (Resistant)	0.52	0.49	-0.50	1.10	20.00	5.70	1.50	0.20
**Equally or better than '93M11' in all experiments**								
PI153282	-0.40	-0.16	-0.66	1.00	20.00	3.50	1.50	-0.03
PI196150	0.09	-0.38	-1.13	1.00	20.00	3.50	1.50	-0.44
PI378679	0.31	-0.16	-0.56	1.00	20.00	5.50	1.50	-0.22
PI467324	-0.46	-0.38	-0.57	1.00	20.00	3.50	1.50	-0.29
PI507491	-0.98	-0.15	-1.26	1.00	20.00	5.00	1.50	-0.72
PI549056	-0.55	-0.38	-0.57	1.00	20.00	4.50	1.50	-0.33
PI567264A	-0.89	-0.38	-0.66	1.00	20.00	3.50	1.50	-0.67
PI594457A	-0.48	0.03	-0.56	1.00	20.00	3.00	1.00	-0.47
PI594902	-0.27	-0.38	-0.57	1.00	20.00	4.50	1.50	-0.39
PI603335B	-0.22	-0.38	-0.56	1.00	20.00	4.50	1.50	-0.35
PI603674	-0.26	-0.15	-0.66	1.00	20.00	4.00	1.00	0.02
**Lowest 10% of disease ranking in both field trials**								
PI086081	-1.49	0.25	0.13	2.00	14.00	7.00	3.00	-0.98
PI086452	-0.84	-0.38	0.13	2.00	5.50	38.00	3.00	-0.83
PI089156	-1.08	-0.16	0.13	1.50	14.00	15.50	2.50	-0.75
PI227212	-1.36	0.25	0.13	2.00	12.00	23.00	3.00	-1.69
PI379561	-0.83	0.23	0.13	0.00	0.00	0.00	0.00	-0.76
PI437156C	-1.30	0.04	0.13	1.50	9.50	24.00	2.50	-0.76

PI437733	-0.93	0.04	0.13	1.00	20.00	3.00	1.50	-0.84
PI458061A	-1.20	0.23	0.13	0.50	20.00	5.00	2.00	-0.93
PI479738	-0.84	-0.38	-0.56	1.00	10.50	16.00	2.50	-1.14
PI504485	-0.81	-0.38	-0.66	1.00	20.00	5.00	2.00	-0.71
PI507491	-0.98	-0.15	-1.26	1.00	20.00	5.00	1.50	-0.72
PI567154	-0.85	-0.38	-0.56	1.00	20.00	8.00	2.00	-0.65
PI567264A	-0.89	-0.38	-0.66	1.00	20.00	3.50	1.50	-0.67
PI578473A	-1.17	-0.15	0.13	1.00	20.00	3.50	1.50	-0.85

14FLD = 2014 field, 14GHSE = 2014 greenhouse, 15GHSE= 2015 greenhouse, and 15FLD=2015 field environments

DAI03, DAI14 = Plant severity score given at 3 and 14 DAI, WS = wilt score, and LL = lesion length

Lower genotypic values correspond to disease resistance for severity (Severity, DAI03, and DAI14) and lesion length (LL) responses and disease susceptibility in wilt score (WS) responses.

Table S3. SNPs significantly associated with soybean SSR resistance through genome-wide association and their predicted candidate genes.

CHR	EXP	Trait	SNP[a]	MAF	P	QTL[b]	Candidate genes[c]	Annotation	Category
5	14FLD	Sev	*ss715590908*	0.16	6.74E-05	N	*Glyma.05g 144500*	Two component response regulator	DNA/RNA
7			*ss715596530*	0.41	4.36E-04	[90]	*Glyma.07g 136900*	Serine/threonine-protein kinase	Signaling
8			*ss715599948*	0.37	9.53E-05	[33,75,91]	*Glyma.08g 215700*	Ethylene response factor(ERF)/AP2 transcription factor family	Signaling
11			*ss715610499*	0.43	3.65E-04	N	*Glyma.11g 254900*	UDP-glucuronate 4-epimerase	Cell wall
12			*ss715612173*	0.14	2.83E-04	N	*Glyma.12g 169300*	7 transmembrane mildew resistance locus O (MLO) family protein	Defense
19			*ss715635935*	0.33	8.52E-05	N	*Glyma.19g 248900*	Ethylene response factor	Signaling
20			*ss715637528*	0.34	3.55E-04	N	*Glyma.20g 109300*	Flavonol 7-O-glucosyltransferase	Secondary Metabolism
1	14GHSE	DAI03	*ss715579141*	0.49	5.93E-05	N	*Glyma.01g 034600*	GDSL/SGNH-like Acyl-Esterase family found in Pmr and Cas1p	Cell wall
4			*ss715588567*	0.06	1.93E-05	N	*Glyma.04g 209700*	Lipase (class 3)	Defense
8			*ss715601247*	0.38	4.47E-05	N	*Glyma.08g 033200*	Multi antimicrobial extrusion (MatE) protein	Defense
2	14GHSE	DAI14	*ss715581133*	0.06	2.57E-06	N	*Glyma.02g 013900*	Myeloblastosis (myb)-like DNA binding protein	Secondary metabolism
3			*ss715584553*	0.34	1.92E-05	N	*Glyma.03g 001500*	Syringolide-induced protein	Defense
9			*ss715603406*	0.31	8.44E-06	[90,92]	*Glyma.09g 119600*	LRR-RLK with Toll-like receptor	Membrane
9			*ss715603408*	0.30	5.86E-06	[90,92]	*Glyma.09g 120100*	EMG1/NEP1 methlytransferase	RNA/DNA
9			*ss715603485*	0.29	3.61E-05	[92]	*Glyma.09g 126900*	Voltage dependent anion channel	Membrane
9			*ss715603491*	0.29	5.36E-05	[92]	*Glyma.09g 127300*	Isoflavone 7-O-glucosyltransferase 1-like	Secondary metabolism

9			*ss715603503*	0.28	6.06E-05	92	*Glyma.09g 127800*	Adenine phosphoribosyl transferase	Primary metabolism
9			*ss715604491*	0.13	5.26E-05	N	*Glyma.09g 048400*	Peroxidase	Oxidation
9			*ss715604590*	0.20	9.18E-05	N	*Glyma.09g 049100*	Cytochrome P450	Miscellaneous
9			*ss715604731*	0.08	5.77E-05	N	*Glyma.09g 051100*	Cellulose synthase	Cell wall
9			*ss715604981*	0.11	6.28E-05	90	*Glyma.09g 052900*	ERF/AP2 transcription factor family	Signaling
9			*ss715605054*	0.17	1.25E-04	90	*Glyma.09g 053800*	Ankyrin repeat family protein	Miscellaneous
12	15GHSE	DAI03	*ss715612209*	0.12	2.55E-04	N	*Glyma.12g 171300*	7 transmembrane MLO family protein	Defense
14			*ss715618815*	0.44	1.70E-04	91	*Glyma.14g 172200*	Protein containing VQ motif	Unknown
17			*ss715628199*	0.38	1.95E-04	33	*Glyma.17g 097900*	WRKY DNA-binding protein	Defense
18			*ss715630238*	0.15	4.88E-05	N	*Glyma.18g 039400*	LRR-RLK	Signaling
18			*ss715630264*	0.18	4.20E-06	N	*Glyma.18g 039900*	Unknown	Protein
18			*ss715632705*	0.33	2.63E-04	N	*Glyma.18g 082100*	Flavonol 7-O-glucosyltransferase	Defense
20			*ss715638266*	0.39	1.44E-04	N	*Glyma.20g 184200*	Myb-like DNA-binding domain	DNA/RNA
20			*ss715638269*	0.40	1.32E-04	N	*Glyma.20g 185100*	LRR-RLK, RNI-like	Signaling
2	15GHSE	WS	*ss715581558*	0.35	6.65E-05	N	*Glyma.02g 026200*	NB-ARC domain-containing disease resistance protein	Defense
6			*ss715595168*	0.19	7.54E-05	N	*Glyma.06g 314000*	Ethylene insensitive 3	Signaling
7			*ss715596502*	0.39	2.36E-04	90	*Glyma.07g 135400*	LRR-RLK, legume lectin domain	Signaling
10			*ss715607699*	0.09	8.73E-05	33	*Glyma.10g 247900*	Cysteine Synthase	Protein
16			*ss715624738*	0.39	9.09E-05	N	*Glyma.16g 188700*	Disease resistance family protein / LRR-RLK with Toll-like receptor	Defense
18			*ss715631537*	0.34	1.49E-04	N	*Glyma.18g*	Peroxidase	Oxidation

							211100		
19			*ss715634826*	0.36	3.34E-04	33	*Glyma.19g 134100*	LRR and NB-ARC domain-containing disease resistance protein	Defense
1	15GHSE	LL	*ss715578907*	0.11	1.59E-05	N	*Glyma.01g 028000*	Leucine-rich repeat receptor-like protein kinase (LRR-RPK) with salt-stress response/antifungal domain	Signaling
10			*ss715607699*	0.09	1.69E-06	33	*Glyma.10g 247900*	Cysteine Synthase	Protein
10			*ss715608353*	0.25	6.76E-05	N	*Glyma.10g 063500*	Tetratricopeptide repeat (TPR)-like superfamily protein	Unknown
11			*ss715608756*	0.42	2.39E-04	N	*Glyma.11g 001300*	LRR-RLK	Signaling
11			*ss715611260*	0.06	8.70E-05	N	*Glyma.11g 012900*	Aspartyl protease	Defense
13			*ss715614675*	0.30	7.28E-05	33	*Glyma.13g 172400*	Negative regulator of systemic acquired resistance (SAR)	Defense
14			*ss715619067*	0.10	1.00E-04	91	*Glyma.14g 187200*	Ubiquitin fold modifier-specific peptidase	Protein
14			*ss715619085*	0.11	1.23E-04	91	*Glyma.14g 188400*	Pathogenesis-related thaumatin superfamily protein	Defense
19			*ss715633818*	0.10	2.38E-04	N	Unknown	…	…
19			*ss715634536*	0.06	3.74E-05	N	*Glyma.19g 115000*	LRR-RLK, BRI1-like	Signaling
1	15GHSE	DAI14	*ss715578851*	0.14	2.47E-04	N	*Glyma.01g 086200*	RNA recognition motif	DNA/RNA
7			*ss715596502*	0.39	6.10E-05	90	*Glyma.07g 135400*	LRR-RLK, legume lectin domain	Signaling
7			*ss715596517*	0.23	7.99E-05	90	*Glyma.07g 136600*	Serine/threonine-protein kinase	Signaling
19			*ss715633601*	0.26	2.42E-04	N	*Glyma.19g 077800*	Cytosol aminopeptidase	Protein
2	15FLD	Sev	*ss715583594*	0.23	7.71E-05	N	*Glyma.02g 059700*	LRR-RLK with LysM domain	Signaling
13			*ss715613722*	0.17	1.45E-04	N	*Glyma.13g 036800*	GTPase	Signaling
13			*ss715615422*	0.20	3.49E-04	75	*Glyma.13g 080200*	Glycosyl hydrolase	Cell wall
13			*ss715616351*	0.42	3.10E-04	N	*Glyma.13g 328100*	Small ubiquitin-like modifier (SUMO) ligase	Defense

14			*ss715618320*	0.17	1.21E-04	[92]	*Glyma.14g 042400*	LRR-RLK putative disease resistant protein induced by chitin oligomers	Defense
14			*ss715618415*	0.17	9.34E-05	[92]	*Glyma.14g 044400*	LRR-RLK	Signaling
15			*ss715621805*	0.36	3.54E-04	N	*Glyma.15g 221600*	AP2/ethylene reponseive element binding protein (EREBP) transcription factor	Signaling
16			*ss715623502*	0.33	1.27E-04	N	*Glyma.16g 017700*	Basic helix-loop-helix (bHLH) DNA-binding superfamily	DNA/RNA
16			*ss715625404*	0.32	1.36E-04	N	*Glyma.16g 072000*	2-oxoglutarate (2OG) and Fe(II)-dependent oxygenase	Oxidation

14FLD = 2014 field, 14GHSE = 2014 greenhouse, 15GHSE= 2015 greenhouse, and 15FLD=2015 field environments

DAI03, DAI14 = Plant severity score given at 3 and 14 DAI, WS = wilt score, and LL = lesion length

CHR=chromosome, EXP=Experiment,

[a]SNP ID is assigned on SoyBase[12].

[b]QTL previously reported or newly identified in the present study, N: QTLs have not been previously reported.

[c]Genes annotated in Glyma1.1, Glyma1.0, and NCBI RefSeq gene models in SoyBase[12] served as the source of candidate genes.

Table S4. SNP-SNP interactions associated with soybean SSR resistance at high significance threshold in field environments through genome-wide epistasis analysis and their predicted candidate genes.

EXP	CHR$_1$	SNP$_1$[a]	MAF$_1$	Candidate Gene$_1$[b]	Annotation$_1$	Category$_1$	CHR$_2$	SNP$_2$[a]	MAF$_2$	Candidate Gene$_2$[b]	Annotation$_2$	Category$_2$	P-value
14FLD	1	*ss715579233*	0.48	*Glyma.01g036100*	NB-ARC domain-containing disease resistance protein	Defense	14	*ss715618500*	0.31	*Glyma.14g047300*	Unknown	Unknown	9.06E-21
	1	*ss715579249*	0.46	*Glyma.01g036600*	C2H2 zinc finger protein	DNA/RNA	14	*ss715618500*	0.31	*Glyma.14g047300*	Unknown	Unknown	4.84E-21
	4	*ss715589210*	0.36	*Glyma.04g083700*	Exostosin-related protein	Misc.	12	*ss715613101*	0.47	*Glyma.12g062700*	Expansin	Cell wall	6.25E-21
	6	*ss715593089*	0.30	*Glyma.06g164900*	Auxin response factor	Signaling	16	*ss715624192*	0.44	*Glyma.16g141000*	Sugar Transporter	Membrane	3.10E-20
	6	*ss715593089*	0.30	*Glyma.06g164900*	Auxin response factor	Signaling	16	*ss715624203*	0.45	*Glyma.16g141700*	Geranylgeranyl pyrophosphate synthase	Secondary metabolism	5.20E-20
	6	*ss715593129*	0.30	*Glyma.06g166800*	SWEET sucrose efflux transporter	Membrane	16	*ss715624158*	0.44	*Glyma.16g138800*	Vacuolar glucose exporter	Membrane	8.28E-20
	6	*ss715593129*	0.30	*Glyma.06g166800*	SWEET sucrose efflux transporter	Membrane	16	*ss715624181*	0.44	*Glyma.16g139700*	Clathrin adaptor complex	Membrane	1.01E-19
	6	*ss715593129*	0.30	*Glyma.06g166800*	SWEET sucrose efflux transporter	Membrane	16	*ss715624192*	0.44	*Glyma.16g141000*	Sugar Transporter	Membrane	1.42E-21
	6	*ss715593129*	0.30	*Glyma.06g166800*	SWEET sucrose efflux transporter	Membrane	16	*ss715624199*	0.42	*Glyma.16g141300*	GRAS family of transcription factors	DNA/RNA	1.02E-20
	6	*ss715593129*	0.30	*Glyma.06g166800*	SWEET sucrose efflux transporter	Membrane	16	*ss715624203*	0.45	*Glyma.16g141700*	Geranylgeranyl pyrophosphate synthase	Secondary metabolism	2.42E-21
	10	*ss715605960*	0.28	*Glyma.10g028300*	Sucrose-binding protein	Membrane	10	*ss715605970*	0.50	*Glyma.10g028700*	Methylcrotonyl-CoA carboxylase (MCCase) biotin containing subunit	Primary metabolism	7.24E-20
	11	*ss715611069*	0.42	*Glyma.11g094000*	Unknown	Unknown	13	*ss715616735*	0.46	*Glyma.13g067900*	Glucosyl transferase	Cell wall	1.27E-21
	11	*ss715611069*	0.42	*Glyma.11g094000*	Unknown	Unknown	16	*ss715624211*	0.27	*Glyma.16g142400*	Pectate lyase	Cell wall	1.56E-19

	12	*ss715613081*	0.33	*Glyma.12g058900*	RAB GTPase	Signaling	12	*ss715613082*	0.36	*Glyma.12g059200*	Senescence-associated gene	Senescence	7.20E-21
	14	*ss715617886*	0.27	*Glyma.14g024500*	Disease resistance protein, Toll-Interleukin receptor (TIR)-nucleotide-binding site (NBS)-LRR	Defense	19	*ss715635906*	0.34	*Glyma.19g036100*	UDP-glucosyl transferase	Cell wall	6.33E-21
	14	*ss715617924*	0.29	*Glyma.14g026200*	GDSL/SGNH-like Acyl-Esterase family found in Pmr and Cas1p	Cell wall	19	*ss715635906*	0.34	*Glyma.19g036100*	UDP-glucosyl transferase	Cell wall	3.65E-20
	14	*ss715617374*	0.18	*Glyma.14g102000*	Endomembrane protein	Membrane	18	*ss715628872*	0.30	*Glyma.18g018400*	Ethylene insensitive 3	Signaling	1.03E-20
	19	*ss715636458* *ss715636461*	0.44	*Glyma.19g053000*	Arogenate dehydratase	Primary metabolism	19	*ss715633286*	0.47	Unknown	…	…	2.94E-20
	19	*ss715636458* *ss715636461*	0.44	*Glyma.19g053000*	Arogenate dehydratase	Primary metabolism	19	*ss715636503*	0.45	*Glyma.19g054300*	Amino acid transporter	Membrane	1.48E-20
	19	*ss715634141*	0.33	*Glyma.19g098500*	Senescence-related gene 3	Senescence	19	*ss715634188*	0.31	*Glyma.19g100800*	LRR-RLK, phytosulfokin receptor 1	Signaling	3.04E-21
	19	*ss715634188*	0.31	*Glyma.19g100800*	LRR-RLK, phytosulfokin receptor 1	Signaling	19	*ss715634227*	0.32	*Glyma.19g103000*	E3 ubiquitin ligase	Protein	1.10E-19
	19	*ss715634204*	0.31	*Glyma.19g101600*	Nuclear cap-binding protein	Signaling	19	*ss715634219*	0.34	*Glyma.19g102800*	UDP-glucosyl transferase	Primary metabolism	4.81E-20
15FLD	3	*ss715586788*	0.40	*Glyma.03g040900*	Late upregulated in response to Hyaloperonospora parasitica (LURP)-1	Defense	11	*ss715611267*	0.28	*Glyma.11g116600*	Glucose-1-phosphate adenylyltransferase	Primary metabolism	1.31E-19
	15	*ss715622572*	0.43	*Glyma.15g064900*	LRR-RLK, S-locus lectin protein family	Signaling	15	*ss715622587*	0.20	*Glyma.15g064900*	LRR-RLK, S-locus lectin protein family	Signaling	3.10E-20

14FLD = 2014 field, 15FLD=2015 field environments

CHR=chromosome, EXP=Experiment, MAF= minor allele frequency

[a]SNP ID is assigned on SoyBase[12].

[b]Genes annotated in Glyma1.1, Glyma1.0, and NCBI RefSeq gene models in SoyBase[12] served as the source of candidate genes.

Table S5. Accessions used from the USDA Soybean Germplasm Core Collection, their country of origin and maturity group based on USDA GRIN data, and genotypic values for Sclerotinia stem rot resistance in multiple greenhouse and field environments for multiple traits.

	14FLD	14GHSE		15GHSE				15FLD	Maturity[a]	Country of Origin[a]
Accession	Severity	DAI03	DAI14	DAI03	WS	LL	DAI14	Severity		
92Y83	0.581	0.985	0.461	1.30	15.70	27.40	2.10	0.567	…	…
93M11	0.517	0.486	-0.496	1.10	20.00	5.70	1.50	0.199	…	…
AXN1_55	…	…	…	…	…	…	…	-0.236	…	…
PI054591	0.330	0.035	0.126	2.00	5.50	52.00	3.00	-0.256	III	Unknown
PI054608_1	0.066	0.461	0.126	1.00	20.00	7.50	2.00	-0.090	II	China
PI054854	0.553	0.031	0.126	1.00	20.00	3.50	1.50	-0.210	I	China
PI062202	-0.132	0.045	0.126	1.00	20.00	7.50	2.00	-0.478	III	China
PI065388	1.402	…	…	2.00	14.50	25.00	2.50	0.847	II	China
PI068685	0.463	0.035	0.126	1.00	7.50	26.00	3.00	-0.254	II	China
PI068788	0.981	-0.222	0.064	1.50	17.00	19.00	2.50	0.701	II	China
PI068815	0.965	-0.222	0.064	1.50	20.00	8.00	2.00	-0.143	II	Unknown
PI070241	0.595	-0.152	0.126	1.50	12.50	30.50	2.50	0.076	I	China
PI070463	-0.241	0.247	0.126	1.50	6.50	34.50	3.00	-0.386	II	China
PI071161	0.295	0.045	0.126	1.00	20.00	5.00	1.00	0.681	I	China
PI072232	-0.084	0.045	0.126	1.50	12.00	48.50	2.50	-0.294	III	China
PI079586	1.188	0.031	0.126	1.50	12.00	41.50	2.50	0.774	II	China
PI079593	0.537	0.247	0.126	1.00	20.00	1.00	1.00	1.324	II	China
PI079648	0.777	0.461	0.126	2.00	4.00	63.00	3.00	-0.119	I	China
PI079691_4	1.186	0.461	0.126	1.50	20.00	5.00	2.00	0.681	III	Unknown
PI079694	0.892	0.035	0.126	1.50	12.50	24.00	2.50	-0.219	I	Unknown
PI079727	0.191	-0.284	-0.658	1.50	20.00	6.50	2.00	0.006	I	Unknown
PI079756	0.514	-0.152	0.126	1.50	13.00	50.50	2.00	0.558	II	China
PI079870_1	0.851	0.247	0.126	1.50	15.00	14.00	2.50	-0.116	I	China

PI080459	-1.046	-0.152	0.126	1.50	12.00	43.00	2.50	-0.065	III	Japan
PI080461	0.259	-0.165	0.126	2.00	5.00	76.00	3.00	0.371	III	Unknown
PI080469	0.359	0.247	0.126	1.50	20.00	4.50	1.50	0.189	II	Unknown
PI080831	-0.308	0.045	0.126	1.00	20.00	5.50	1.00	0.133	III	China
PI081044_2	0.319	-0.562	-0.660	2.00	6.00	88.00	3.00	0.014	III	Unknown
PI081667	-0.581	-0.152	0.126	1.00	20.00	1.00	1.00	0.012	III	Unknown
PI081763	0.074	0.461	0.126	2.50	4.50	47.50	3.00	0.205	II	China
PI081765	0.893	0.035	-0.660	2.00	5.50	52.00	3.00	1.004	I	China
PI081766	0.416	0.035	-0.660	2.00	6.50	47.00	3.00	-0.210	III	China
PI081767	0.272	0.241	0.064	2.00	4.00	70.00	3.00	0.454	II	China
PI081768	-0.163	0.035	0.126	1.00	4.00	38.50	3.00	-0.811	II	China
PI081770	0.659	0.231	0.126	2.00	13.00	28.50	2.50	0.668	II	China
PI081771	0.564	0.035	0.126	2.00	6.50	36.50	3.00	0.073	II	China
PI081773	1.651	-0.222	0.064	2.00	4.50	67.50	3.00	0.188	II	Unknown
PI082278	-0.883	…	…	…	…	…	…	-0.247	III	South Korea
PI084611	-0.189	0.035	0.126	1.00	7.00	40.00	3.00	-0.359	III	South Korea
PI084921	0.212	0.231	0.126	1.50	13.50	28.00	2.50	0.716	II	Unknown
PI084973	-1.092	-0.152	0.126	1.00	20.00	4.00	2.00	-0.350	III	Japan
PI085009_1	-0.525	-0.152	0.126	2.50	11.50	35.50	2.50	0.080	III	Japan
PI085356	-0.799	-0.165	0.126	1.00	13.50	20.00	2.50	-0.402	III	Unknown
PI086002	0.910	-0.222	0.064	2.00	13.50	17.50	2.50	0.538	II	Unknown
PI086006	0.548	0.247	0.126	2.00	6.50	52.00	3.00	0.444	III	Japan
PI086046	0.305	0.031	0.126	2.00	6.00	34.50	3.00	0.473	II	Japan
PI086081	-1.488	0.247	0.126	2.00	14.00	7.00	3.00	-0.983	III	Japan
PI086145	-0.258	0.231	0.126	1.50	12.00	55.00	2.50	-0.924	III	Japan
PI086449	0.411	-0.222	-0.781	2.00	5.50	80.50	3.00	0.513	III	Unknown
PI086452	-0.845	-0.384	0.126	2.00	5.50	38.00	3.00	-0.827	III	Japan
PI087600_1	-1.462	-0.284	-0.658	1.00	8.50	29.00	3.00	0.843	III	Unknown
PI087618	0.442	-0.165	0.126	1.50	20.00	5.50	2.00	-0.108	III	North Korea
PI087631_1	-0.602	-0.384	-0.660	2.00	6.50	69.50	3.00	0.299	III	Unknown
PI087634	-0.150	-0.384	0.126	1.00	20.00	3.00	1.00	-0.246	III	Unknown

PI088289	0.179	0.035	0.126	1.00	20.00	10.50	2.00	0.031	III	China
PI088292	-0.235	0.247	0.126	1.50	17.00	10.00	2.50	-0.317	III	China
PI088294_1	0.309	-0.152	0.126	1.50	14.00	13.50	2.00	0.366	II	China
PI088295	1.032	-0.222	0.064	1.50	9.50	10.00	2.50	0.091	I	China
PI088305	0.207	-0.152	0.126	2.00	7.00	42.00	3.00	0.702	III	China
PI088306	0.149	-0.152	0.126	1.50	14.50	17.00	2.50	0.197	III	China
PI088497	-0.319	-0.165	-0.560	1.50	12.50	22.00	2.00	-0.070	I	China
PI088788	1.608	0.247	0.126	1.50	12.00	39.00	2.00	0.411	III	China
PI089003_1	-0.914	0.231	0.126	2.00	17.00	17.50	2.50	0.238	II	Unknown
PI089008	-0.131	0.031	-0.658	1.00	20.00	6.50	2.00	-0.179	II	China
PI089060	0.101	0.231	0.126	1.00	20.00	4.00	2.00	-0.277	I	Unknown
PI089130	-0.198	-0.152	0.126	1.00	20.00	2.50	1.00	-0.044	III	Unknown
PI089134	-0.726	0.045	0.126	2.00	6.50	40.50	3.00	0.226	III	North Korea
PI089152	0.194	0.231	0.126	1.50	13.00	34.00	2.50	-0.380	III	Unknown
PI089153	-0.829	-0.384	0.126	2.00	7.00	57.00	3.00	0.261	II	North Korea
PI089154	0.250	0.247	0.126	2.00	6.00	49.50	3.00	0.041	II	North Korea
PI089156	-1.079	-0.165	0.126	1.50	14.00	15.50	2.50	-0.748	II	North Korea
PI089773	-0.312	-0.152	0.126	1.50	4.50	88.50	3.00	-0.053	III	China
PI090392	0.739	-0.165	0.126	2.00	5.00	79.50	3.00	-0.147	III	China
PI091091	0.301	0.035	-0.660	1.00	13.50	24.50	2.00	0.028	II	China
PI091102	0.117	0.241	0.064	1.50	13.00	51.50	2.50	0.135	II	Unknown
PI091120_3	0.871	0.035	0.126	1.50	8.00	18.00	3.00	-0.067	III	Unknown
PI091162	-0.539	0.461	0.126	1.50	12.50	25.00	2.50	-0.888	III	Unknown
PI091341	-0.375	0.247	0.126	1.50	12.00	39.00	2.50	0.836	III	China
PI091349	0.070	0.247	0.126	1.50	12.00	56.50	2.00	-0.768	III	China
PI091559	0.168	0.031	0.126	1.50	12.50	48.00	2.50	0.120	II	China
PI091725_3	-0.276	0.231	0.126	1.50	13.50	21.00	2.00	-0.691	II	Unknown
PI091733	1.389	0.045	-0.560	1.50	13.50	21.00	2.00	-0.852	I	China
PI092465	0.574	0.247	0.126	2.00	14.00	17.00	2.50	0.072	II	Russia
PI092603	-0.482	0.035	0.126	1.50	12.00	54.00	2.00	-0.004	II	China
PI092611	-0.621	0.045	0.126	1.50	9.00	37.00	3.00	-0.416	II	China

PI092683	-0.569	-0.542	-0.658	1.50	13.00	17.00	2.50	0.070	II	China
PI092706	-0.649	0.035	0.126	2.00	5.50	49.00	3.00	-0.335	I	China
PI096162	-0.399	0.461	0.126	2.00	13.50	9.00	2.50	-0.552	II	Unknown
PI096199	0.222	0.035	0.126	1.50	12.50	34.00	2.00	-0.506	III	Unknown
PI096322	0.429	0.043	0.070	1.50	17.00	7.50	2.50	-0.143	III	North Korea
PI096786_1	-0.083	0.247	0.126	1.00	7.00	47.50	3.00	0.521	III	Unknown
PI131531	-0.474	0.247	0.126	1.00	20.00	4.00	1.50	-0.402	I	Poland
PI135589	0.429	…	…	1.50	14.00	17.00	2.50	0.189	II	China
PI135590	1.465	0.247	0.126	2.00	6.00	72.50	3.00	0.782	II	China
PI153229	-0.326	0.461	0.126	1.50	17.00	9.00	2.50	0.350	I	France
PI153250	-0.590	-0.165	0.126	1.00	20.00	3.00	1.50	-0.776	I	Belgium
PI153280	-0.664	-0.152	0.126	1.00	20.00	6.50	2.00	0.490	II	France
PI153282	-0.400	-0.165	-0.660	1.00	20.00	3.50	1.50	-0.031	I	Belgium
PI157421	-0.225	0.461	0.126	1.00	20.00	5.50	2.00	0.331	III	South Korea
PI167240	-0.842	-0.384	0.126	1.00	20.00	6.00	2.00	-0.367	III	Turkey
PI171450	-1.281	0.031	0.126	1.50	20.00	5.50	1.00	-0.245	III	Japan
PI173994	-0.817	-0.152	0.126	2.00	20.00	13.00	2.00	0.079	III	South Korea
PI181536	0.730	-0.384	0.126	2.00	5.00	58.50	3.00	-0.959	I	Japan
PI181537	0.216	-0.152	0.126	1.00	20.00	4.50	2.00	-0.420	II	Japan
PI189916	0.367	-0.152	-0.658	1.00	20.00	6.00	2.00	-0.384	I	China
PI189919	-0.296	-0.165	0.126	1.00	20.00	5.50	2.00	-0.076	I	France
PI189930	-0.177	0.247	0.126	1.50	9.50	25.00	2.50	0.185	II	France
PI189941	-0.699	0.035	0.126	1.00	20.00	5.00	2.00	-0.063	I	France
PI189958	-0.405	-0.384	0.126	2.00	13.50	16.00	2.50	-0.632	II	France
PI189962	-0.169	-0.152	0.126	2.00	8.00	22.00	3.00	…	I	France
PI189969	0.658	0.247	0.126	1.50	17.00	13.00	2.50	-0.183	III	France
PI196149	-0.669	0.045	0.126	1.00	20.00	8.00	1.50	-0.112	III	Japan
PI196150	0.088	-0.384	-1.125	1.00	20.00	3.50	1.50	-0.444	II	Japan
PI200478	0.293	0.031	0.126	1.50	12.00	41.50	2.50	-0.802	III	Japan
PI200548	-0.243	-0.165	-0.660	1.50	13.50	29.00	2.00	-0.336	III	Japan
PI205085	-0.699	-0.152	0.126	2.00	20.00	7.00	2.00	-0.856	I	Japan

PI205087	-0.037	-0.111	0.126	2.00	11.50	47.50	2.50	-1.334	III	Japan
PI227212	-1.360	0.247	0.126	2.00	12.00	23.00	3.00	-1.685	III	Japan
PI227325	-0.263	-0.384	0.126	1.00	20.00	2.00	1.00	-0.038	I	Japan
PI227558	0.329	-0.152	0.126	1.00	20.00	5.00	1.50	-0.891	II	Japan
PI229336	-1.233	0.035	0.126	1.50	12.50	32.50	2.50	0.263	III	Japan
PI232987	1.338	0.031	0.126	2.00	6.50	35.50	3.00	0.274	II	China
PI232988	0.463	…	…	2.50	4.50	50.00	3.00	0.354	II	China
PI232989	0.826	0.031	-0.565	2.00	6.00	35.00	3.00	-0.531	II	China
PI232990	1.263	…	…	1.00	20.00	5.00	2.00	1.225	II	China
PI232992	-0.164	-0.384	-0.658	2.00	14.00	43.50	2.50	0.440	III	Japan
PI248509A	-0.719	0.045	0.126	1.50	20.00	5.00	2.00	0.154	I	China
PI250844	-0.696	0.045	0.126	1.00	20.00	4.00	2.00	-0.297	I	Iran
PI253650A	-0.333	-0.165	0.126	1.50	12.00	27.00	2.50	0.009	II	China
PI253651C	1.117	0.231	0.126	2.00	6.00	35.50	3.00	1.156	III	China
PI253652C	0.440	-0.384	-0.660	1.50	20.00	2.50	1.50	-0.235	I	China
PI253653D	0.958	-0.383	0.064	2.00	17.00	10.50	2.50	0.550	I	China
PI253658A	-0.504	0.031	0.126	1.50	8.00	20.00	3.00	-0.121	I	China
PI253660B	-0.417	0.045	0.126	1.50	14.00	8.00	3.00	0.093	III	China
PI261466	-0.890	-0.165	0.126	2.00	20.00	10.00	2.00	0.621	III	Japan
PI261474	0.092	-0.384	-1.416	1.00	20.00	1.50	1.00	0.601	II	China
PI266806A	-0.540	-0.152	0.126	2.00	11.50	27.50	3.00	0.937	II	China
PI290149	0.325	-0.384	-1.416	1.00	13.50	16.50	2.50	-0.619	I	Hungary
PI291274B	0.630	0.260	0.070	1.00	8.00	27.00	3.00	0.869	I	China
PI291275	0.110	…	…	1.00	20.00	6.00	2.00	-0.701	I	China
PI291276	0.386	-0.384	-0.658	1.50	4.50	119.50	3.00	0.723	I	China
PI291277	0.325	-0.384	-0.658	2.00	7.00	28.50	3.00	-0.076	I	China
PI291278	0.426	0.461	0.126	1.00	20.00	3.00	1.00	0.576	I	China
PI291309C	-0.490	-0.200	0.070	2.00	5.50	31.00	3.00	0.800	I	China
PI297538	0.363	-0.384	-1.125	2.00	14.50	8.50	2.50	-0.095	I	Hungary
PI323586B	0.277	0.461	0.126	1.00	20.00	3.50	1.00	-0.128	II	Portugal
PI326579	0.415	…	…	3.00	3.00	71.00	3.00	-0.271	I	Romania

PI326580	0.404	-0.315	-0.660	1.00	20.00	3.00	2.00	0.783	I	Germany
PI339868E	0.400	0.461	0.126	2.00	13.50	20.00	2.50	-0.358	III	South Korea
PI361080	0.379	0.461	0.126	1.50	20.00	5.00	2.00	-0.068	II	Russia
PI361090	-0.710	0.461	0.126	1.50	20.00	3.50	2.00	0.456	I	Austria
PI361101	-0.114	-0.165	0.126	2.00	6.00	36.00	3.00	0.620	III	Korea
PI378663	-0.295	0.247	0.126	1.50	6.00	45.00	3.00	1.060	I	Russia
PI378679	0.309	-0.165	-0.560	1.00	20.00	5.50	1.50	-0.220	I	France
PI379559D	-1.123	…	…	1.00	20.00	4.00	2.00	-0.296	III	Japan
PI379561	-0.830	0.231	0.126	…	…	…	…	-0.759	III	Japan
PI391577	-0.139	0.461	0.126	1.00	20.00	2.00	1.00	0.092	II	China
PI391586	0.555	-0.152	0.126	2.00	7.00	31.00	3.00	0.153	III	China
PI398813	-1.307	0.035	0.126	1.50	20.00	3.50	1.50	-0.145	III	South Korea
PI398881	-0.153	-0.152	0.126	1.50	12.00	29.50	2.50	-0.681	III	South Korea
PI401418	0.863	-0.152	-0.658	2.00	6.00	74.00	3.00	0.434	I	Russia
PI404160B	0.235	-0.152	0.126	1.00	20.00	9.00	2.00	-0.173	III	Georgia
PI404166	1.040	0.461	0.126	2.00	20.00	8.50	2.00	0.084	III	China
PI404169B	-0.353	-0.152	-0.565	1.50	20.00	8.00	2.00	0.309	III	China
PI407653	0.405	-0.222	0.064	1.00	20.00	3.00	2.00	-0.455	III	China
PI407656	0.351	0.461	0.126	1.00	20.00	7.00	2.00	0.414	II	China
PI407659A	0.263	-0.384	0.126	3.00	3.00	58.50	3.00	0.420	II	China
PI407746	0.718	0.231	0.126	2.00	6.50	46.50	3.00	0.426	III	China
PI407810	0.045	0.461	0.126	2.50	5.00	55.50	3.00	0.511	III	South Korea
PI416762	-0.221	0.035	0.126	2.00	7.50	19.00	3.00	-0.042	II	Japan
PI416773	-0.890	0.461	0.126	2.00	5.50	51.50	3.00	-0.160	II	Japan
PI416835	0.554	-0.200	0.070	2.00	20.00	6.00	2.00	0.738	II	Japan
PI416868A	-0.144	-0.384	0.126	1.00	20.00	4.00	1.50	-0.196	III	Japan
PI416892	-0.097	0.031	-0.565	1.50	13.00	15.50	2.50	-0.147	III	Japan
PI417054	0.536	-0.384	0.126	1.50	12.00	25.50	2.00	-1.843	III	Japan
PI417091	0.649	0.045	0.126	1.50	20.00	5.50	1.50	-0.553	II	Japan
PI417138	1.150	…	…	2.00	5.00	38.50	3.00	-0.150	II	Japan
PI417139	0.689	0.461	0.126	1.50	4.50	40.00	3.00	1.167	I	Japan

PI417167	-0.796	0.035	0.126	1.00	9.00	4.00	2.50	-1.141	III	Japan
PI417198	0.295	0.247	0.126	2.00	20.00	7.00	2.00	-0.297	III	Japan
PI417297	0.636	-0.165	-0.660	1.50	14.00	9.00	3.00	-0.571	III	Japan
PI417513B	-0.305	-0.384	-0.658	2.00	12.00	39.00	2.50	0.067	I	Eastern Europe
PI417517	-0.445	0.461	0.126	1.00	13.00	25.50	2.50	0.056	I	Yugoslavia
PI417559	-0.350	-0.165	0.126	2.00	5.50	46.00	3.00	-0.822	III	Poland
PI423870	0.255	-0.384	-1.262	1.50	20.00	7.50	2.00	-0.603	II	Japan
PI424005	-0.266	…	…	…	…	…	…	0.326	III	South Korea
PI424078	0.095	…	…	2.00	5.50	47.50	3.00	-0.422	III	South Korea
PI430596	-0.912	0.045	0.126	2.00	20.00	6.50	2.00	0.059	II	China
PI430597	-0.251	-0.152	0.126	1.00	20.00	2.50	1.50	-0.165	II	China
PI430619	0.768	-0.165	0.126	1.00	20.00	7.50	2.00	0.099	III	China
PI437091	0.187	0.461	0.126	1.50	12.50	15.00	2.50	-0.296	I	Russia
PI437098	-0.334	-0.384	-0.560	1.00	20.00	6.00	2.00	-0.580	I	Russia
PI437116	0.791	0.241	0.064	2.00	7.00	35.00	3.00	1.638	I	Russia
PI437121B	0.333	-0.152	-0.565	2.00	5.50	77.50	3.00	0.332	II	Russia
PI437122	-0.075	0.231	0.126	2.00	7.50	38.00	3.00	-0.308	II	Russia
PI437124	0.657	0.035	0.126	1.50	14.00	15.00	2.50	0.549	III	Georgia
PI437145B	0.064	0.461	0.126	1.00	7.00	29.00	3.00	0.066	II	Russia
PI437156C	-1.298	0.045	0.126	1.50	9.50	24.00	2.50	-0.758	I	Russia
PI437165A	0.295	-0.152	-0.658	1.50	6.00	60.50	3.00	0.256	I	Russia
PI437174B	-0.540	-0.384	0.126	1.00	20.00	5.00	2.00	-0.333	I	Russia
PI437340B	-0.043	0.031	0.126	1.00	20.00	4.00	1.50	-0.988	II	Russia
PI437343	-0.302	0.231	0.126	1.00	12.00	5.50	2.00	0.575	I	Russia
PI437345	-0.054	0.031	0.126	2.00	4.00	71.50	3.00	1.057	II	Russia
PI437356	-0.269	0.461	0.126	1.00	20.00	7.50	2.00	-0.108	II	Russia
PI437377	0.667	0.231	0.126	1.00	20.00	6.00	1.50	0.242	III	Russia
PI437399	-0.307	0.461	0.126	1.50	12.00	35.50	2.50	-0.344	II	Russia
PI437425	-0.005	-0.152	0.126	1.00	20.00	3.00	1.50	0.025	I	Russia
PI437427B	-0.693	-0.384	0.126	1.50	13.00	17.50	2.00	-0.578	II	Russia
PI437462A	0.061	0.231	0.126	1.50	20.00	3.50	1.50	0.023	II	Russia

PI437477A	-0.225	0.035	0.126	1.50	6.00	54.00	3.00	0.039	I	Russia
PI437509	-0.141	-0.152	0.126	1.00	20.00	5.00	2.00	-0.462	I	Russia
PI437519	-0.541	0.045	0.126	1.00	9.00	11.00	3.00	-0.520	I	Russia
PI437558	0.282	…	…	2.00	20.00	10.00	2.00	0.456	I	China
PI437581	-0.568	0.461	0.126	2.00	7.00	50.50	3.00	-0.047	II	China
PI437585	-0.986	-0.384	0.126	2.00	6.00	44.00	3.00	0.014	II	China
PI437592	-0.031	0.045	0.126	1.00	20.00	6.00	2.00	-0.471	II	China
PI437594A	0.039	0.035	0.126	2.00	20.00	7.50	2.00	0.953	I	China
PI437651B	-0.365	-0.152	-0.658	1.00	20.00	5.00	1.00	0.407	II	China
PI437656	0.233	0.241	0.064	1.00	20.00	3.00	2.00	0.907	II	China
PI437662	0.400	…	…	2.00	20.00	8.00	2.00	-0.077	II	China
PI437663	1.078	0.461	0.126	2.00	13.50	28.00	2.50	0.276	II	China
PI437674	0.558	0.035	0.126	1.00	10.00	6.00	3.00	-0.226	III	China
PI437682A	-0.445	-0.384	0.126	1.00	20.00	4.00	1.50	0.339	I	China
PI437690	0.158	-0.165	0.126	1.00	20.00	4.00	2.00	0.098	III	China
PI437715	0.591	0.035	0.126	1.00	20.00	5.00	2.00	0.297	II	China
PI437716A	-0.899	0.247	0.126	1.00	13.50	13.00	2.50	0.205	I	China
PI437733	-0.929	0.045	0.126	1.00	20.00	3.00	1.50	-0.839	I	China
PI437738B	0.184	0.045	0.126	1.50	10.50	24.00	3.00	0.285	I	China
PI437757	0.157	-0.152	0.126	1.50	14.00	8.50	2.50	-0.025	I	China
PI437786	0.446	-0.384	-0.660	2.00	4.00	72.50	3.00	0.367	I	China
PI437803	-0.113	0.035	0.126	1.00	20.00	7.00	2.00	-0.859	II	China
PI437840A	1.618	0.241	0.064	3.00	3.00	74.00	3.00	0.349	II	China
PI437846	0.372	0.043	0.070	1.50	20.00	9.00	2.00	-0.206	I	China
PI437935	0.699	0.031	0.126	2.00	10.00	22.00	3.00	0.348	II	China
PI437944	0.422	-0.152	0.126	1.00	20.00	5.00	2.00	0.748	II	China
PI437949	-0.380	0.461	0.126	1.00	20.00	4.50	2.00	0.424	I	China
PI437950	0.720	0.035	0.126	1.00	20.00	2.00	1.00	0.585	II	China
PI437964A	-0.025	0.231	0.126	2.00	6.50	30.00	3.00	0.387	II	China
PI437973	0.586	0.031	0.126	1.00	17.00	11.00	2.00	0.208	II	China
PI438031	0.358	0.231	0.126	1.00	20.00	3.00	1.00	0.388	I	China

PI438094B	-0.140	0.247	0.126	1.00	7.00	51.00	3.00	-0.300	I	China
PI438103	1.033	0.247	0.126	1.00	20.00	6.50	2.00	-0.278	II	China
PI438133B	-0.245	-0.384	0.126	1.00	20.00	5.00	1.00	-0.004	II	China
PI438139	-0.464	0.035	0.126	2.00	13.50	14.00	2.50	0.107	II	China
PI438152	-0.024	0.035	0.126	1.50	13.00	21.00	2.50	0.472	II	China
PI438173	-0.109	-0.152	0.126	2.00	7.00	31.00	3.00	0.804	II	China
PI438194	1.114	-0.152	0.126	1.00	20.00	3.00	1.50	-0.330	II	China
PI438218	-1.147	-0.562	-0.735	1.50	12.00	28.00	2.50	0.094	I	China
PI438259B	1.259	-0.384	0.126	1.50	13.50	20.50	2.50	0.895	III	China
PI438292	0.162	0.231	0.126	1.00	20.00	5.00	2.00	-0.324	I	Japan
PI438312	0.060	0.035	0.126	1.50	13.00	30.50	2.50	0.743	III	Algeria
PI438376	0.288	0.247	0.126	2.00	5.50	53.00	3.00	-0.035	I	France
PI438434	0.448	0.247	0.126	2.00	6.00	58.00	3.00	0.108	II	Morocco
PI438503A	0.218	-0.111	-0.660	2.00	20.00	9.00	2.00	0.537	II	United States
PI445819	0.252	…	…	1.00	20.00	4.00	2.00	0.046	I	Germany
PI445845	-0.162	-0.152	0.126	1.50	14.50	11.50	2.50	-0.040	III	China
PI458052	1.139	-0.165	0.126	1.00	20.00	3.00	1.50	-0.321	III	South Korea
PI458061A	-1.197	0.231	0.126	0.50	20.00	5.00	2.00	-0.933	III	South Korea
PI458110	0.184	-0.562	-0.560	2.00	5.50	57.50	3.00	0.137	III	South Korea
PI458307A	-1.132	0.035	0.126	1.50	20.00	5.00	1.50	0.316	III	South Korea
PI458506	-0.023	0.045	0.126	2.50	4.00	61.00	3.00	-0.219	II	China
PI458507	0.992	0.461	0.126	1.00	20.00	3.00	1.00	0.307	III	China
PI458517	0.628	0.045	0.126	1.00	20.00	5.00	2.00	0.242	III	China
PI458519A	1.229	0.231	0.126	1.50	12.00	39.50	2.50	1.406	II	China
PI458520	-0.515	0.247	0.126	1.50	13.00	22.00	2.50	0.214	II	China
PI458521	0.613	-0.165	0.126	1.50	13.50	12.50	2.00	0.759	III	China
PI458522	0.630	0.231	0.126	2.00	4.00	81.50	3.00	0.537	II	China
PI458825B	-0.167	-0.284	0.126	1.50	5.00	72.00	3.00	-0.483	I	China
PI461509	0.762	-0.380	0.070	1.50	13.00	39.00	2.50	-0.346	I	China
PI464877	0.126	0.031	0.126	1.50	9.00	43.00	3.00	0.122	III	China
PI464878	0.800	-0.384	-0.658	1.00	20.00	9.00	2.00	0.645	II	China

PI464880	-0.102	0.045	0.126	2.00	5.00	55.50	3.00	0.219	II	China
PI464884	-0.208	-0.562	-0.735	1.50	20.00	5.00	1.50	-0.116	II	China
PI464914B	-0.545	0.247	0.126	1.50	12.50	27.00	2.50	-0.287	III	China
PI464915A	1.271	0.247	0.126	2.00	5.00	96.50	3.00	1.090	II	China
PI467307	-0.862	-0.165	0.126	1.50	12.50	30.50	2.50	-0.383	I	China
PI467310	0.583	0.461	0.126	2.00	13.50	28.50	2.50	0.480	II	China
PI467311A	-0.322	-0.152	0.126	1.50	13.50	23.00	2.50	0.345	I	China
PI467312	-0.251	0.461	0.126	1.50	14.00	20.00	3.00	-0.069	II	China
PI467324	-0.464	-0.384	-0.565	1.00	20.00	3.50	1.50	-0.285	I	China
PI467327	0.721	0.241	0.064	1.00	20.00	7.00	2.00	0.043	II	China
PI467328	-0.217	-0.165	0.126	2.00	12.00	38.50	2.50	0.449	I	China
PI467332	0.718	0.247	0.126	1.00	20.00	5.00	2.00	0.670	II	China
PI467334B	-0.601	0.035	0.126	1.50	14.50	17.00	2.50	0.216	II	China
PI468381	-0.552	0.045	0.126	1.00	10.00	15.00	3.00	-0.901	II	Japan
PI468384	-0.761	-0.152	0.126	2.00	20.00	6.50	2.00	-0.056	III	China
PI468385	0.651	-0.384	-0.658	2.00	20.00	8.50	2.00	-0.125	III	China
PI468907	0.192	0.035	-0.560	1.00	20.00	4.00	2.00	-0.124	I	China
PI468914	1.020	0.045	0.126	2.00	8.50	26.50	3.00	0.752	III	China
PI468919	-0.919	-0.200	0.070	…	…	…	…	0.450	III	China
PI470223	-0.748	-0.165	-0.660	2.00	13.50	17.00	2.50	-0.378	II	China
PI470227B	-0.035	0.247	0.126	1.50	7.00	28.00	3.00	-0.443	III	China
PI471899	-0.858	-0.152	-0.658	2.00	7.00	34.00	3.00	0.857	III	Indonesia
PI475810	-0.003	0.035	-0.660	2.00	5.50	61.00	3.00	-0.518	II	China
PI475811B	-0.622	0.031	0.126	2.00	4.00	92.00	3.00	-0.552	II	China
PI475818	-0.359	0.045	0.126	1.50	12.00	54.50	2.50	0.425	III	China
PI475820	-0.446	0.247	0.126	2.00	12.50	45.50	2.50	-0.711	II	China
PI475822B	0.756	-0.165	-0.660	1.00	20.00	2.50	1.50	0.401	III	China
PI476344	-0.057	-0.384	-0.660	1.00	11.50	16.00	3.00	-0.337	II	Uzbekistan
PI476345	-0.635	-0.152	0.126	1.00	20.00	4.00	1.50	-0.237	I	Moldova
PI476348	-0.578	0.035	0.126	1.00	20.00	4.50	2.00	0.216	I	Ukraine
PI476911	-0.023	0.247	0.126	1.00	14.00	13.00	3.00	0.188	II	Vietnam

PI479711	0.026	-0.165	-0.560	2.00	4.00	88.00	3.00	-0.239	II	China
PI479713	-0.454	-0.152	0.126	2.00	13.50	22.50	2.50	0.221	II	China
PI479718B	0.247	0.247	0.126	2.00	5.00	75.50	3.00	0.218	II	China
PI479719	-0.297	-0.384	-0.560	1.00	20.00	6.00	2.00	-0.179	I	China
PI479724A	-1.146	-0.384	-0.660	2.00	6.00	22.00	3.00	-0.018	II	China
PI479729	-1.121	0.231	0.126	1.50	13.00	24.50	2.00	-0.604	III	China
PI479738	-0.843	-0.384	-0.560	1.00	10.50	16.00	2.50	-1.138	II	China
PI479740	0.437	0.045	0.126	1.50	13.50	35.50	2.50	0.343	III	China
PI483459	0.632	0.461	0.126	2.00	12.00	35.50	2.50	0.582	I	China
PI504485	-0.814	-0.384	-0.658	1.00	20.00	5.00	2.00	-0.714	I	Japan
PI504490	0.404	-0.165	0.126	1.00	10.00	16.50	2.50	0.039	II	Taiwan
PI504497	-0.225	0.045	0.126	1.50	20.00	6.50	2.00	-0.184	II	Taiwan
PI506527	-0.639	-0.152	0.126	1.50	14.00	17.00	2.50	0.020	III	Japan
PI506528	0.105	0.247	0.126	2.00	5.00	50.50	3.00	-0.310	III	Japan
PI506529	-0.366	-0.562	-0.660	1.50	12.50	34.00	2.50	-0.700	III	Japan
PI506634	0.675	0.461	0.126	1.50	14.50	10.00	2.50	-0.580	II	Japan
PI506678	1.604	0.031	-0.565	2.00	8.00	10.00	3.00	0.351	I	Japan
PI506800B	-0.553	-0.562	-0.660	1.00	14.00	7.50	3.00	-0.372	III	Japan
PI506887	-0.385	0.461	0.126	2.00	5.00	41.50	3.00	-0.594	III	Japan
PI507027	1.033	0.260	0.070	2.00	12.00	29.50	2.50	1.389	II	Japan
PI507147	-0.523	-0.384	-0.565	1.50	20.00	3.50	1.50	-0.376	III	Japan
PI507171	-0.798	0.461	0.126	1.00	20.00	4.00	1.50	-0.478	III	Japan
PI507195	-0.704	0.461	0.126	1.50	13.00	27.50	2.00	-0.366	II	Japan
PI507267	-1.349	-0.152	0.126	1.50	13.00	26.50	2.00	-0.274	III	Japan
PI507487	-0.528	-0.152	0.126	2.00	6.50	51.50	3.00	-0.069	III	Japan
PI507491	-0.977	-0.152	-1.262	1.00	20.00	5.00	1.50	-0.725	III	Japan
PI507717	-0.561	0.241	0.064	2.00	13.00	13.00	2.50	-0.795	I	North Korea
PI512322C	0.111	0.031	0.126	1.50	12.50	36.00	2.50	0.405	I	Georgia
PI518283	0.702	0.461	0.126	1.50	12.00	37.00	2.50	0.759	II	Taiwan
PI518706A	-0.607	0.035	0.126	1.00	20.00	6.50	2.00	-0.646	I	China
PI518757	-0.296	0.461	0.126	1.00	14.00	14.00	2.00	-0.218	III	Taiwan

PI522188A	-0.298	0.231	0.126	2.00	5.00	68.50	3.00	0.484	I	Russia
PI524994	-0.831	-0.152	0.126	1.00	20.00	1.50	1.00	-0.440	I	Russia
PI532456	-0.499	0.035	0.126	1.00	20.00	4.50	1.50	-0.512	II	China
PI532462A	2.238	-0.152	0.126	2.00	7.00	60.50	3.00	0.756	III	China
PI532472	-0.278	-0.152	0.126	1.00	20.00	3.00	1.50	-0.164	II	Japan
PI538377	-0.436	-0.384	-0.565	2.00	7.50	33.50	3.00	-1.551	III	China
PI538389	0.218	0.461	0.126	2.00	9.00	38.00	3.00	0.759	III	Japan
PI538393	-0.788	0.035	0.126	1.00	17.00	7.00	2.00	-0.066	I	China
PI538400	0.991	0.031	0.126	1.00	20.00	8.50	2.00	0.034	II	China
PI538403	-0.117	-0.222	0.064	1.00	20.00	5.00	2.00	0.366	I	Japan
PI538410B	0.888	-0.152	0.126	1.00	20.00	2.50	1.50	-0.289	I	Japan
PI540739	0.117	0.045	-0.560	1.50	20.00	9.00	2.00	-0.747	I	China
PI548316	0.210	…	…	1.50	12.00	43.00	2.50	0.685	III	China
PI548329	-0.362	-0.384	0.126	1.00	20.00	1.50	1.00	-0.481	I	Japan
PI548336	1.112	0.045	0.126	2.00	13.00	17.00	2.50	-0.021	I	Russia
PI548349	1.208	0.461	0.126	2.00	4.50	56.00	3.00	1.349	III	North Korea
PI548373	0.101	0.231	0.126	2.00	5.00	75.00	3.00	0.280	III	China
PI549021A	-0.378	-0.384	0.126	2.00	12.00	14.50	2.50	-0.936	III	China
PI549031	0.818	-0.222	0.064	1.50	9.50	61.50	3.00	0.386	III	China
PI549041A	0.556	0.461	0.126	1.50	13.00	65.50	2.00	0.301	III	China
PI549056	-0.551	-0.384	-0.565	1.00	20.00	4.50	1.50	-0.333	II	Japan
PI549058	0.419	0.035	0.126	1.00	20.00	6.50	2.00	-0.445	II	Japan
PI549064	-0.263	-0.384	-0.560	1.00	20.00	7.50	2.00	-0.054	II	Japan
PI561227	-0.388	-0.384	-0.735	1.00	9.50	19.00	3.00	-0.177	II	China
PI561230	-0.651	0.035	0.126	1.00	9.00	24.00	3.00	0.293	II	China
PI561232	-0.054	-0.165	0.126	2.00	20.00	7.00	2.00	-0.255	I	China
PI561315	-0.113	-0.384	0.126	1.50	12.50	24.50	2.50	-0.462	I	China
PI561333	0.841	0.247	0.126	2.00	13.50	22.50	2.50	1.206	I	China
PI561346	-0.531	0.035	0.126	2.00	10.50	17.50	3.00	0.193	I	China
PI561348	-0.489	-0.165	0.126	1.00	20.00	2.00	1.00	-0.460	I	China
PI561349	0.017	-0.111	-0.660	2.00	5.50	39.50	3.00	-0.197	II	China

PI561377	0.224	0.461	0.126	2.00	4.50	72.00	3.00	0.697	II	Japan
PI562387	-0.158	0.043	0.070	1.50	7.00	36.00	3.00	0.754	I	Japan
PI567154	-0.846	-0.384	-0.560	1.00	20.00	8.00	2.00	-0.654	II	Japan
PI567157A	…	…	…	…	…	…	…	-1.682	0	China
PI567159A	-0.562	0.045	0.126	1.00	14.50	13.00	2.50	-0.114	I	China
PI567161	-0.071	-0.152	0.126	1.00	20.00	1.50	1.00	-0.190	II	China
PI567163	0.542	-0.384	-0.658	1.00	20.00	2.50	1.00	-0.490	I	China
PI567170A	0.568	-0.152	0.126	2.00	11.00	10.50	3.00	-0.628	II	China
PI567170B	0.017	0.461	0.126	1.00	20.00	7.00	2.00	0.531	II	China
PI567214B	0.420	-0.152	0.126	1.50	13.50	36.00	2.50	0.223	I	Russia
PI567223	0.340	-0.384	0.126	2.00	6.00	39.50	3.00	-0.568	I	Russia
PI567229A	1.585	0.247	0.126	1.00	20.00	6.00	2.00	0.743	I	Russia
PI567241	-0.653	0.035	0.126	1.00	20.00	2.50	1.50	0.635	II	China
PI567250B	-0.691	0.461	0.126	1.00	20.00	8.00	2.00	-0.042	III	China
PI567255A	-0.505	-0.152	0.126	1.00	9.00	17.00	3.00	-0.052	I	China
PI567261B	-0.514	-0.562	0.126	1.00	20.00	8.00	2.00	0.102	II	China
PI567262D	-0.559	-0.152	0.126	2.00	7.00	55.50	3.00	0.191	II	China
PI567264A	-0.892	-0.384	-0.658	1.00	20.00	3.50	1.50	-0.665	II	China
PI567266A	-0.685	-0.384	-0.660	2.00	5.50	73.50	3.00	0.501	II	China
PI567267A	-0.625	-0.562	-0.565	1.00	14.50	17.50	2.50	0.077	II	China
PI567275	0.494	0.045	0.126	1.00	20.00	5.00	1.50	-0.664	II	Japan
PI567277	-0.650	-0.384	0.126	1.50	20.00	6.00	2.00	0.072	II	Japan
PI567278	0.129	-0.152	0.126	1.00	17.00	6.00	2.50	-0.489	II	Japan
PI567351A	-0.248	-0.384	0.126	2.00	6.00	82.50	3.00	0.646	II	China
PI567365	-0.593	0.461	0.126	1.00	12.50	34.00	2.50	-0.334	III	China
PI567417B	-0.316	-0.384	-0.560	1.50	14.50	9.00	2.50	0.168	I	China
PI567538B	-0.350	0.035	-0.560	1.00	20.00	5.50	2.00	0.362	II	China
PI567583A	0.172	0.247	0.126	2.00	13.00	27.50	2.50	-0.140	III	China
PI567595A	-0.081	0.461	0.126	1.50	13.00	30.50	2.50	0.238	III	China
PI567619	-0.218	0.260	0.070	1.50	20.00	6.00	1.50	-0.090	III	China
PI567644	-0.428	0.031	0.126	2.00	6.00	96.00	3.00	0.304	III	China

PI567729	-0.338	-0.152	0.126	1.00	20.00	2.00	1.00	-0.189	III	China
PI567774B	-0.223	-0.152	-0.658	2.00	8.00	37.00	3.00	0.218	III	China
PI574478B	-0.542	0.231	0.126	1.50	13.00	21.00	2.50	0.054	II	China
PI574480B	-0.056	-0.384	0.126	2.00	20.00	6.00	2.00	0.331	III	China
PI578360	0.148	0.461	0.126	2.00	20.00	8.00	1.50	0.306	II	China
PI578362	0.594	-0.152	0.126	2.00	10.50	57.50	3.00	0.520	I	China
PI578363	-0.472	-0.384	-0.658	1.50	20.00	12.50	2.00	-0.359	II	China
PI578364	-0.607	-0.284	-0.658	1.50	13.00	26.50	2.00	-0.731	II	China
PI578366	0.111	0.035	0.126	1.00	13.00	3.50	1.00	-0.089	III	China
PI578367	0.773	-0.165	-0.660	2.00	13.50	30.00	2.50	-0.205	III	China
PI578374	0.511	0.035	0.126	2.00	14.00	13.00	2.50	-0.476	I	China
PI578375B	0.063	0.247	0.126	1.00	20.00	2.00	1.00	-0.497	I	China
PI578376	-0.590	-0.165	0.126	2.00	12.00	26.00	2.50	-0.475	II	China
PI578380A	-0.114	-0.384	0.126	1.00	20.00	2.00	1.50	-0.236	I	China
PI578382	-0.207	0.231	0.126	2.00	6.00	51.00	3.00	0.000	I	China
PI578384	0.804	0.031	0.126	2.00	14.50	20.50	2.50	0.031	I	China
PI578385	-0.073	0.461	0.126	1.50	20.00	5.00	1.50	-0.241	I	China
PI578386	-0.130	0.231	0.126	1.00	20.00	5.00	2.00	-0.836	I	China
PI578416	-0.251	0.045	0.126	1.50	20.00	4.50	2.00	-0.031	II	China
PI578439	-1.434	-0.152	-0.658	2.00	20.00	9.00	2.00	0.013	III	Vietnam
PI578473A	-1.173	-0.152	0.126	1.00	20.00	3.50	1.50	-0.847	III	China
PI578474	-0.716	0.461	0.126	2.00	7.00	32.00	3.00	-0.211	I	China
PI578499A	-0.415	0.461	0.126	1.00	13.00	30.50	2.50	-0.052	II	China
PI578499B	0.926	0.035	0.126	2.00	7.00	51.00	3.00	0.505	II	China
PI588008A	-0.583	-0.152	0.126	…	…	…	…	-0.318	III	China
PI592907C	-0.492	0.045	0.126	1.00	20.00	6.50	1.50	0.159	I	Russia
PI592908	0.706	0.461	0.126	1.00	6.50	40.50	3.00	0.003	II	Russia
PI592910	0.648	-0.152	0.126	1.50	12.50	54.50	2.50	0.584	II	Russia
PI592911B	-0.378	0.045	0.126	1.00	20.00	8.50	2.00	0.198	I	Russia
PI592912A	-0.340	-0.165	-0.660	1.50	20.00	4.00	1.50	-0.235	I	Russia
PI592913	0.425	-0.384	0.126	1.00	20.00	10.00	2.00	-0.204	II	Russia

PI593970	-0.341	-0.152	0.126	1.50	13.50	33.50	2.00	0.531	I	Japan
PI593982	1.003	0.260	0.070	2.00	6.50	30.00	3.00	0.654	I	Japan
PI594156	-0.216	0.247	0.126	1.50	20.00	9.00	2.00	-0.638	III	Japan
PI594170B	0.722	0.035	0.126	2.00	9.50	60.50	3.00	-0.451	I	Japan
PI594296	-0.665	0.241	0.064	1.50	13.00	36.50	2.50	-0.582	I	Japan
PI594394	-0.694	-0.165	0.126	2.00	7.00	49.00	3.00	0.400	III	China
PI594457A	-0.480	0.035	-0.560	1.00	20.00	3.00	1.00	-0.465	III	China
PI594471A	-1.284	-0.384	-0.658	1.00	20.00	5.00	2.00	0.013	III	China
PI594898	0.907	-0.165	0.126	1.50	13.00	25.00	2.50	-0.542	I	China
PI594902	-0.272	-0.384	-0.565	1.00	20.00	4.50	1.50	-0.387	I	China
PI597397A	-0.505	-0.111	0.126	1.00	20.00	2.50	1.50	-0.359	I	Russia
PI597405B	-0.533	-0.152	0.126	1.00	20.00	5.00	2.00	-0.635	I	Ukraine
PI597482	0.732	0.031	0.126	1.00	20.00	5.50	2.00	0.617	III	South Korea
PI602497A	0.210	-0.152	-0.658	1.50	17.00	7.00	2.00	-0.019	I	China
PI603151A	-0.940	-0.165	0.126	1.50	20.00	4.00	1.50	-0.193	I	North Korea
PI603334	-0.491	-0.165	0.126	1.00	20.00	4.00	1.50	-0.912	I	China
PI603335B	-0.222	-0.384	-0.560	1.00	20.00	4.50	1.50	-0.351	II	China
PI603337A	0.380	0.461	0.126	1.50	12.00	28.00	2.50	-0.198	I	China
PI603339A	0.262	-0.165	0.126	1.00	20.00	5.50	2.00	-0.082	I	China
PI603367	-0.460	-0.384	0.126	1.00	20.00	5.00	2.00	0.308	I	China
PI603371	0.399	0.247	0.126	2.00	6.50	44.50	3.00	-0.571	I	China
PI603412B	0.712	0.241	0.064	2.00	14.50	13.50	2.50	0.326	II	China
PI603422B	1.008	-0.111	0.126	1.50	20.00	9.00	2.00	0.663	II	China
PI603424C	0.015	0.247	0.126	1.50	14.50	16.00	2.50	0.750	I	China
PI603426F	-0.009	0.031	0.126	1.50	20.00	5.00	2.00	-0.037	I	China
PI603428D	0.454	0.461	0.126	1.00	20.00	6.00	1.50	-0.208	III	China
PI603438E	-0.968	-0.096	-0.658	1.00	20.00	4.00	2.00	0.297	III	China
PI603442	0.441	0.035	-0.560	1.00	20.00	2.50	1.00	0.311	III	China
PI603444A	1.091	0.231	0.126	2.00	5.00	83.50	3.00	-0.564	II	China
PI603452	-0.090	0.031	0.126	2.00	11.00	16.00	3.00	-0.022	III	China
PI603470	0.550	0.247	0.126	1.00	20.00	2.50	1.00	0.334	II	China

PI603546A	0.245	0.231	0.126	1.00	14.50	22.50	2.50	-0.336	I	China
PI603560	-0.308	0.031	0.126	1.50	13.00	20.50	2.50	0.037	III	China
PI603587A	0.057	0.461	0.126	1.00	20.00	2.00	1.00	0.864	I	China
PI603594	-0.470	0.461	0.126	1.00	20.00	3.50	2.00	-0.318	II	China
PI603596	-1.095	0.035	0.126	1.00	20.00	4.50	2.00	0.751	III	China
PI603655	-0.729	-0.384	-0.658	2.00	20.00	6.00	2.00	-0.801	III	China
PI603660	-0.933	-0.152	-0.658	1.00	20.00	5.00	2.00	0.079	II	China
PI603662B	0.397	-0.284	-0.658	1.00	20.00	5.00	1.00	0.802	II	China
PI603674	-0.257	-0.152	-0.660	1.00	20.00	4.00	1.00	0.015	III	China
PI603704A	-0.668	-0.542	-0.658	1.50	12.50	28.00	2.50	0.075	I	China
PI603747	-0.123	-0.152	0.126	1.50	12.00	75.00	2.50	0.136	II	China
PI603749	-0.607	0.035	0.126	1.00	20.00	1.00	1.00	0.007	II	China
PI603912	-0.729	0.045	0.126	…	…	…	…	-0.792	III	North Korea
PI603915C	-0.848	0.461	0.126	1.00	20.00	2.00	1.00	-0.648	III	North Korea
PI612611	0.742	0.035	0.126	1.00	9.00	17.00	3.00	0.304	III	North Korea
PI612711B	-0.321	0.031	0.126	1.00	20.00	3.50	1.50	-0.847	I	China
PI612752	0.362	0.035	0.126	2.00	20.00	6.00	2.00	1.397	I	China
PI612754	0.345	-0.165	0.126	1.50	12.00	50.00	2.50	0.068	I	China
PI612759C	0.187	0.231	0.126	2.00	4.00	103.00	3.00	0.792	I	China
PI612760	0.512	0.241	0.064	1.50	20.00	4.00	1.50	0.617	I	China

14FLD = 2014 field, 14GHSE = 2014 greenhouse, 15GHSE= 2015 greenhouse, and 15FLD=2015 field environments

DAI03, DAI14 = Plant severity score given at 3 and 14 DAI, WS = wilt score, and LL = lesion length

Lower genotypic values correspond to disease resistance for severity (Severity, DAI03, and DAI14) and lesion length (LL) responses and disease susceptibility in wilt score (WS) responses.

[a]Based on USDA GRIN data available through SoyBase[12]
